# Supplementary material for: Sialic Acid-Binding Immunoglobulin-like Lectin G Promotes Atherosclerosis and Liver Inflammation by Suppressing the Protective Functions of B-1 Cells
Source: Cell Rep. 2016 Mar 3;14(10):2348–61. doi: 10.1016/j.celrep.2016.02.027 (PMC4802221; doi:10.1016/j.celrep.2016.02.027)
Supplement: Document S1. Supplemental Experimental Procedures and Figures S1–S7 [file mmc1.pdf]

Cell Reports, Volume *14*

Supplemental Information

Sialic Acid-Binding Immunoglobulin-like Lectin G Promotes Atherosclerosis and Liver Inflammation by Suppressing the Protective Functions of B-1 Cells

Sabrina Gruber, Tim Hendrikx, Dimitrios Tsiantoulas, Maria Ozsvar-Kozma, Laura Göderle, Ziad Mallat, Joseph L. Witztum, Ronit Shiri-Sverdlov, Lars Nitschke, and Christoph J. Binder

SUPPLEMENTAL INFORMATION

Figure S1.

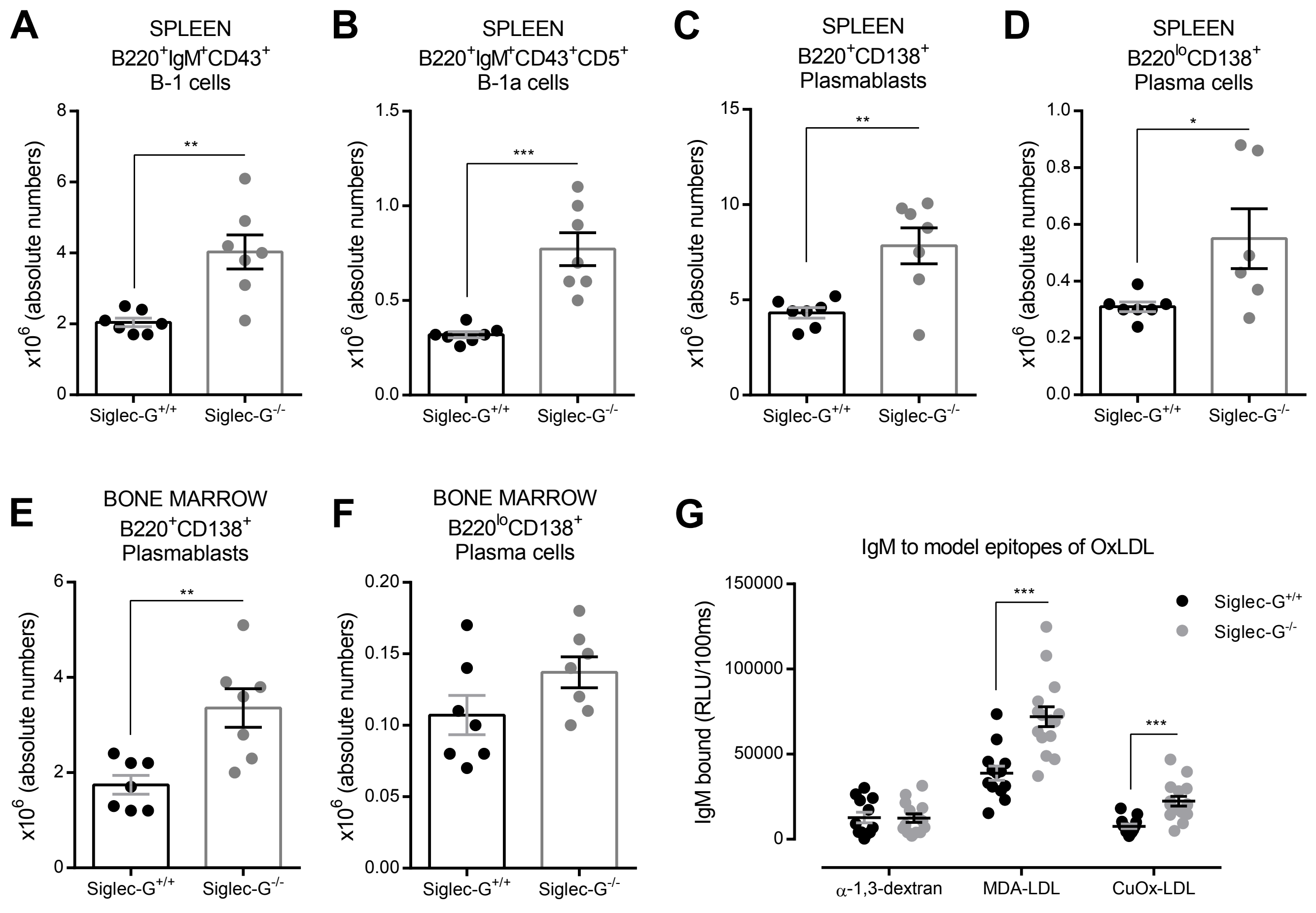

Figure S2.

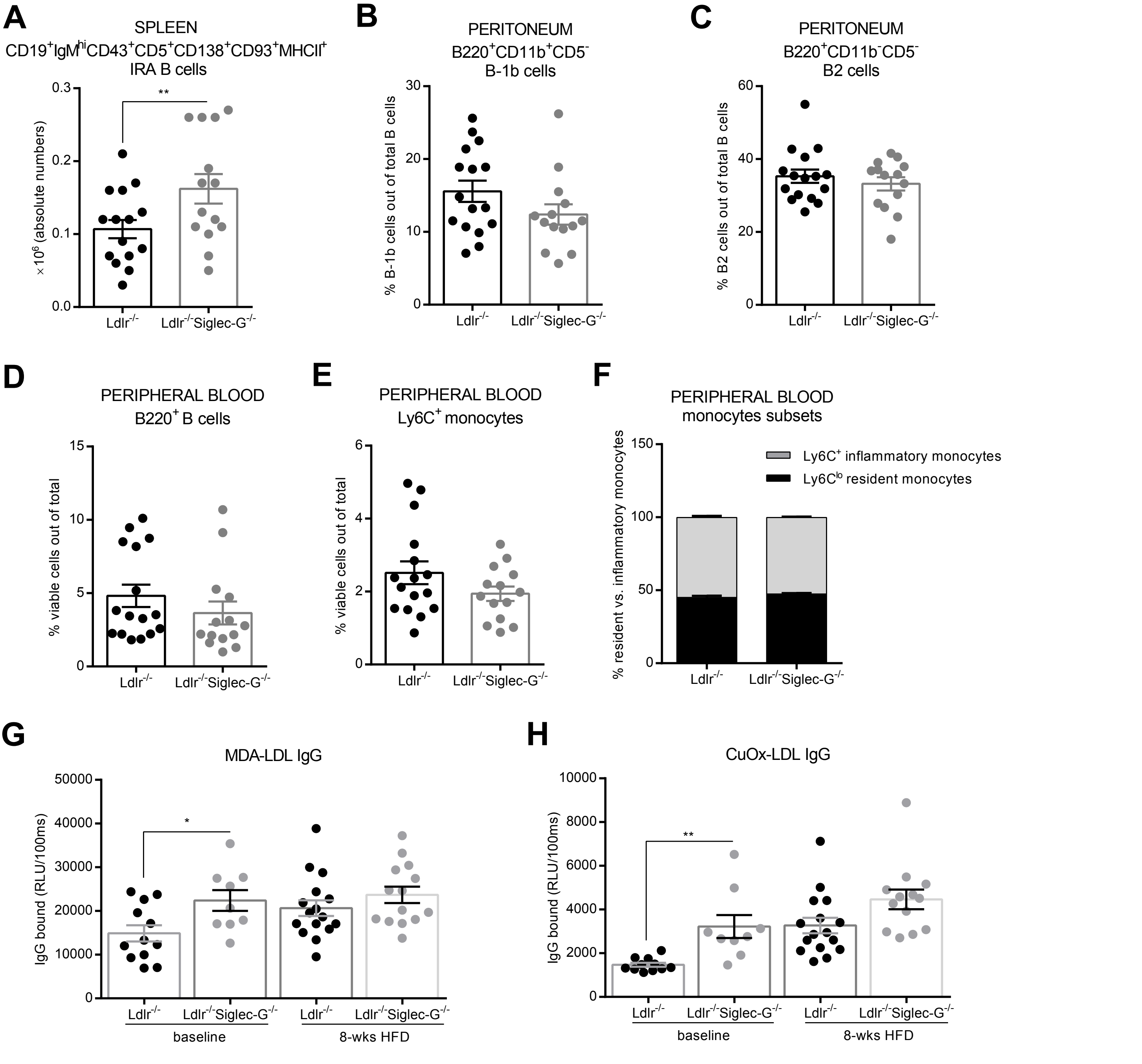
A
SPLEEN
CD19+IgMhiCD43+CD5+CD138+CD93+MHCII+
IRA B cells
×10^6 (absolute numbers)
**
Ldlr-/-
Ldlr-/-Siglec-G-/-
B
PERITONEUM
B220+CD11b+CD5-
B-1b cells
% B-1b cells out of total B cells
C
PERITONEUM
B220+CD11b-CD5-
B2 cells
% B2 cells out of total B cells
D
PERIPHERAL BLOOD
B220+ B cells
% viable cells out of total
E
PERIPHERAL BLOOD
Ly6C+ monocytes
% viable cells out of total
F
PERIPHERAL BLOOD
monocytes subsets
% resident vs. inflammatory monocytes
Ly6C+ inflammatory monocytes
Ly6Clo resident monocytes
G
MDA-LDL IgG
IgG bound (RLU/100ms)
*
baseline
8-wks HFD
H
CuOx-LDL IgG
IgG bound (RLU/100ms)
**
baseline
8-wks HFD

Figure S3.

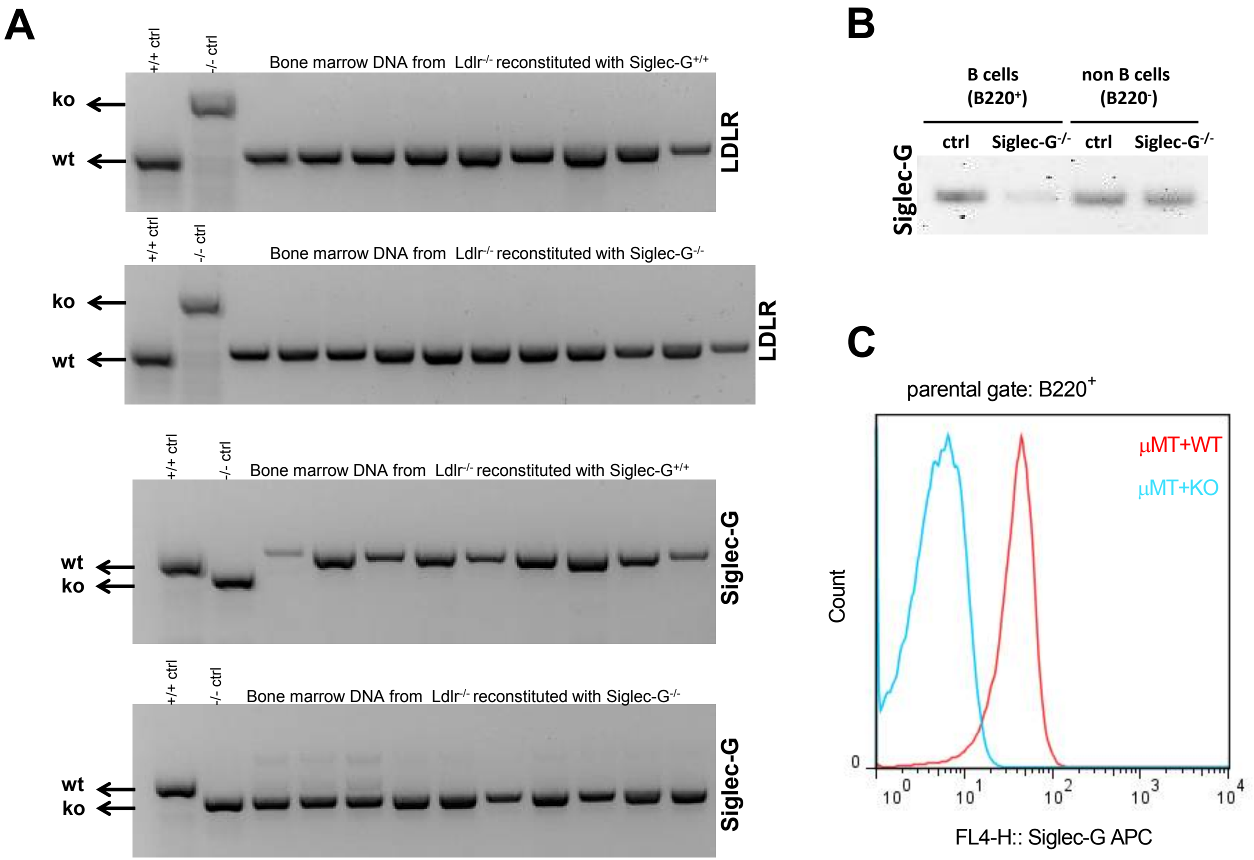
A
+/+ ctrl
-/- ctrl
Bone marrow DNA from Ldlr-/- reconstituted with Siglec-G+/+
ko
wt
LDLR
Bone marrow DNA from Ldlr-/- reconstituted with Siglec-G-/-
Siglec-G
B
B cells (B220+)
non B cells (B220-)
ctrl
Siglec-G-/-
C
parental gate: B220+
μMT+WT
μMT+KO
Count
0
10 0
10 1
10 2
10 3
10 4
FL4-H:: Siglec-G APC

Figure S4.

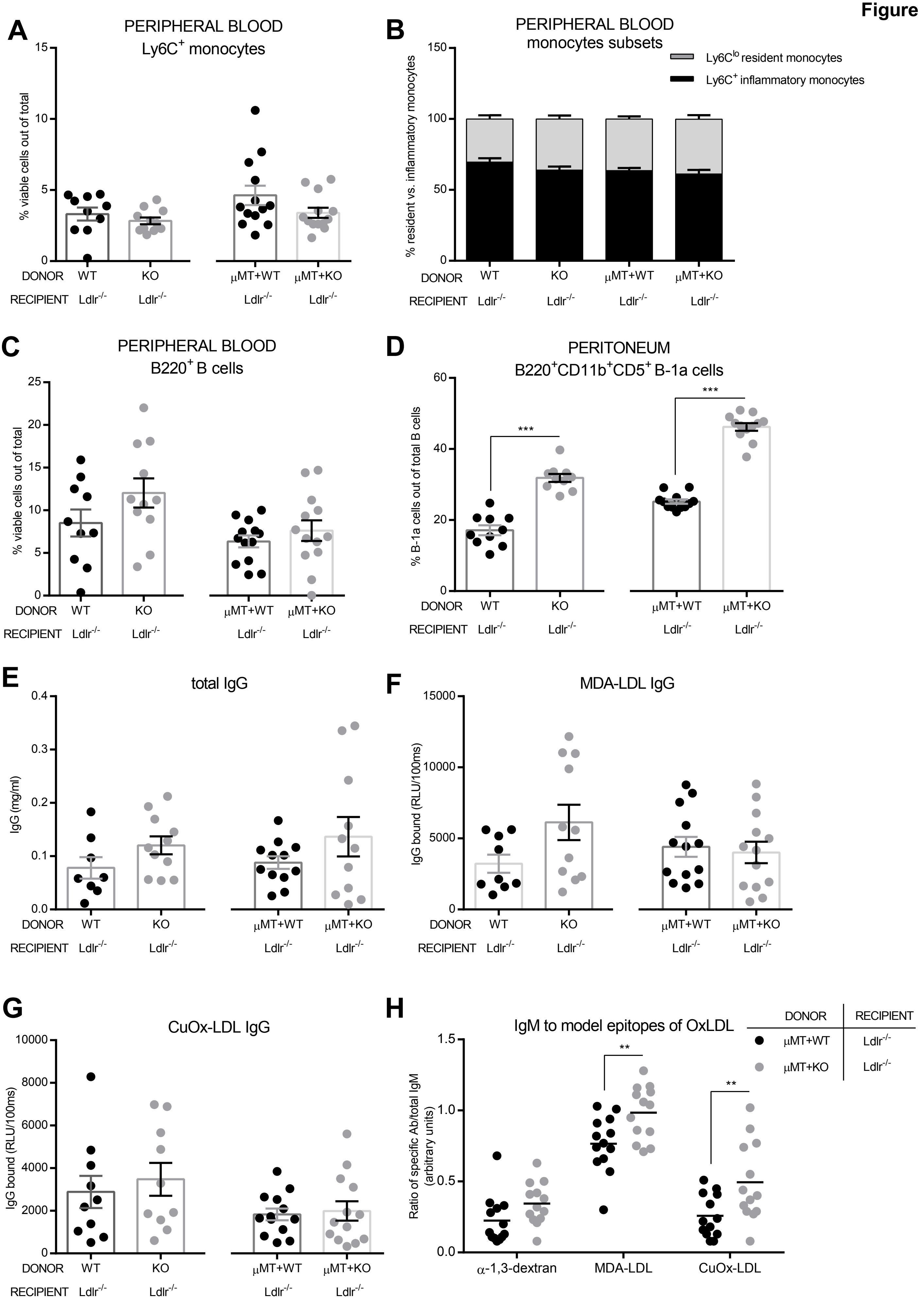
A
PERIPHERAL BLOOD
Ly6C+ monocytes
% viable cells out of total
DONOR WT KO μMT+WT μMT+KO
RECIPIENT Ldlr-/- Ldlr-/- Ldlr-/- Ldlr-/-
B
PERIPHERAL BLOOD
monocytes subsets
Ly6Clo resident monocytes
Ly6C+ inflammatory monocytes
% resident vs. inflammatory monocytes
DONOR WT KO μMT+WT μMT+KO
RECIPIENT Ldlr-/- Ldlr-/- Ldlr-/- Ldlr-/-
C
PERIPHERAL BLOOD
B220+ B cells
% viable cells out of total
DONOR WT KO μMT+WT μMT+KO
RECIPIENT Ldlr-/- Ldlr-/- Ldlr-/- Ldlr-/-
D
PERITONEUM
B220+CD11b+CD5+ B-1a cells
% B-1a cells out of total B cells
***
***
DONOR WT KO μMT+WT μMT+KO
RECIPIENT Ldlr-/- Ldlr-/- Ldlr-/- Ldlr-/-
E
total IgG
IgG (mg/ml)
DONOR WT KO μMT+WT μMT+KO
RECIPIENT Ldlr-/- Ldlr-/- Ldlr-/- Ldlr-/-
F
MDA-LDL IgG
IgG bound (RLU/100ms)
DONOR WT KO μMT+WT μMT+KO
RECIPIENT Ldlr-/- Ldlr-/- Ldlr-/- Ldlr-/-
G
CuOx-LDL IgG
IgG bound (RLU/100ms)
DONOR WT KO μMT+WT μMT+KO
RECIPIENT Ldlr-/- Ldlr-/- Ldlr-/- Ldlr-/-
H
IgM to model epitopes of OxLDL
DONOR RECIPIENT
μMT+WT Ldlr-/-
μMT+KO Ldlr-/-
Ratio of specific Ab/total IgM (arbitrary units)
**
**
α-1,3-dextran MDA-LDL CuOx-LDL

Figure S5.

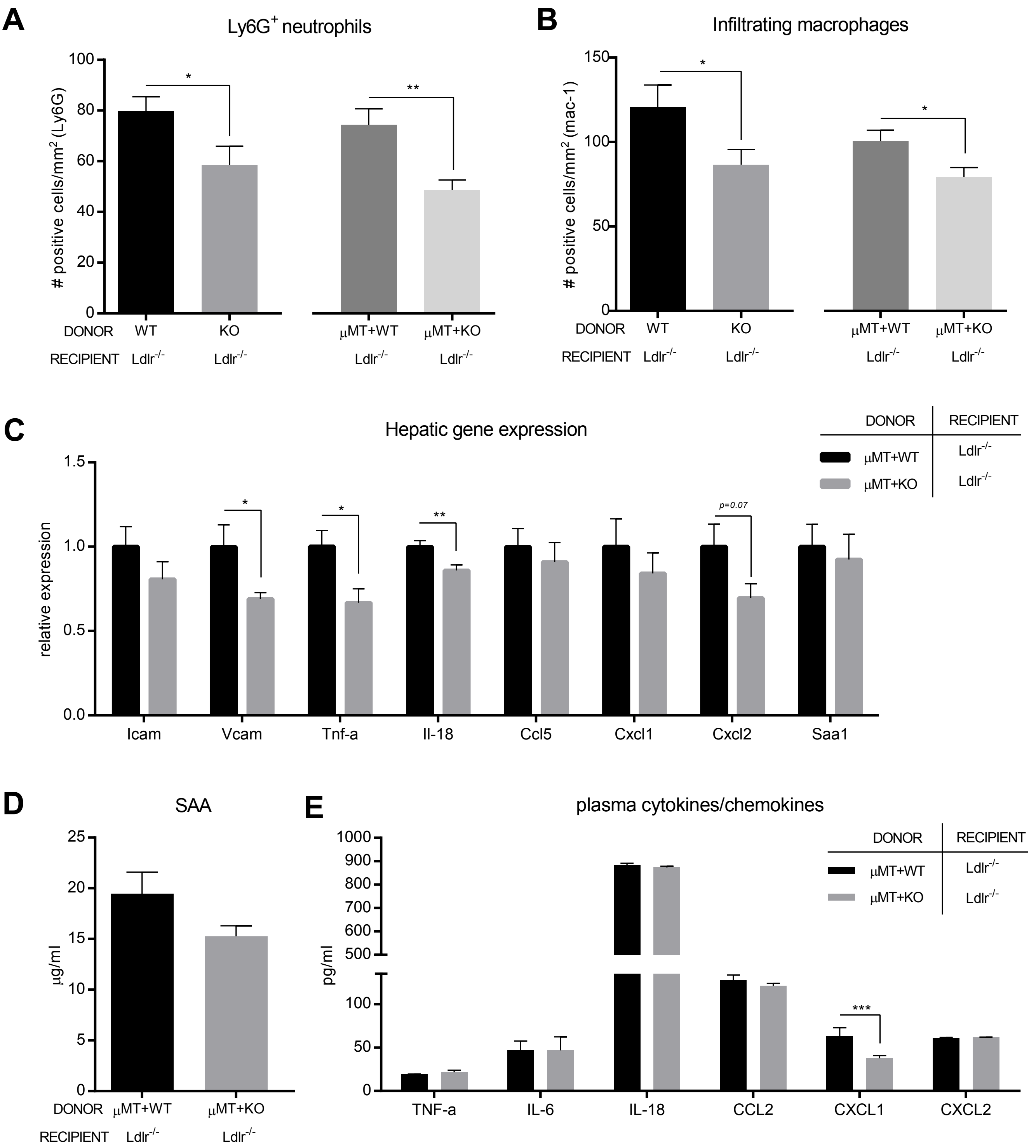
A
Ly6G+ neutrophils
positive cells/mm2 (Ly6G)
0
20
40
60
80
100
*
**
DONOR
WT
KO
μMT+WT
μMT+KO
RECIPIENT
Ldlr-/-
Ldlr-/-
Ldlr-/-
Ldlr-/-
B
Infiltrating macrophages
positive cells/mm2 (mac-1)
0
50
100
150
*
*
DONOR
WT
KO
μMT+WT
μMT+KO
RECIPIENT
Ldlr-/-
Ldlr-/-
Ldlr-/-
Ldlr-/-
C
Hepatic gene expression
relative expression
0.0
0.5
1.0
1.5
*
*
**
p=0.07
Icam
Vcam
Tnf-a
Il-18
Ccl5
Cxcl1
Cxcl2
Saa1
DONOR
RECIPIENT
μMT+WT
Ldlr-/-
μMT+KO
Ldlr-/-
D
SAA
μg/ml
0
5
10
15
20
25
DONOR
μMT+WT
μMT+KO
RECIPIENT
Ldlr-/-
Ldlr-/-
E
plasma cytokines/chemokines
pg/ml
0
50
100
500
600
700
800
900
1000
***
TNF-a
IL-6
IL-18
CCL2
CXCL1
CXCL2
DONOR
RECIPIENT
μMT+WT
Ldlr-/-
μMT+KO
Ldlr-/-

Figure S6.

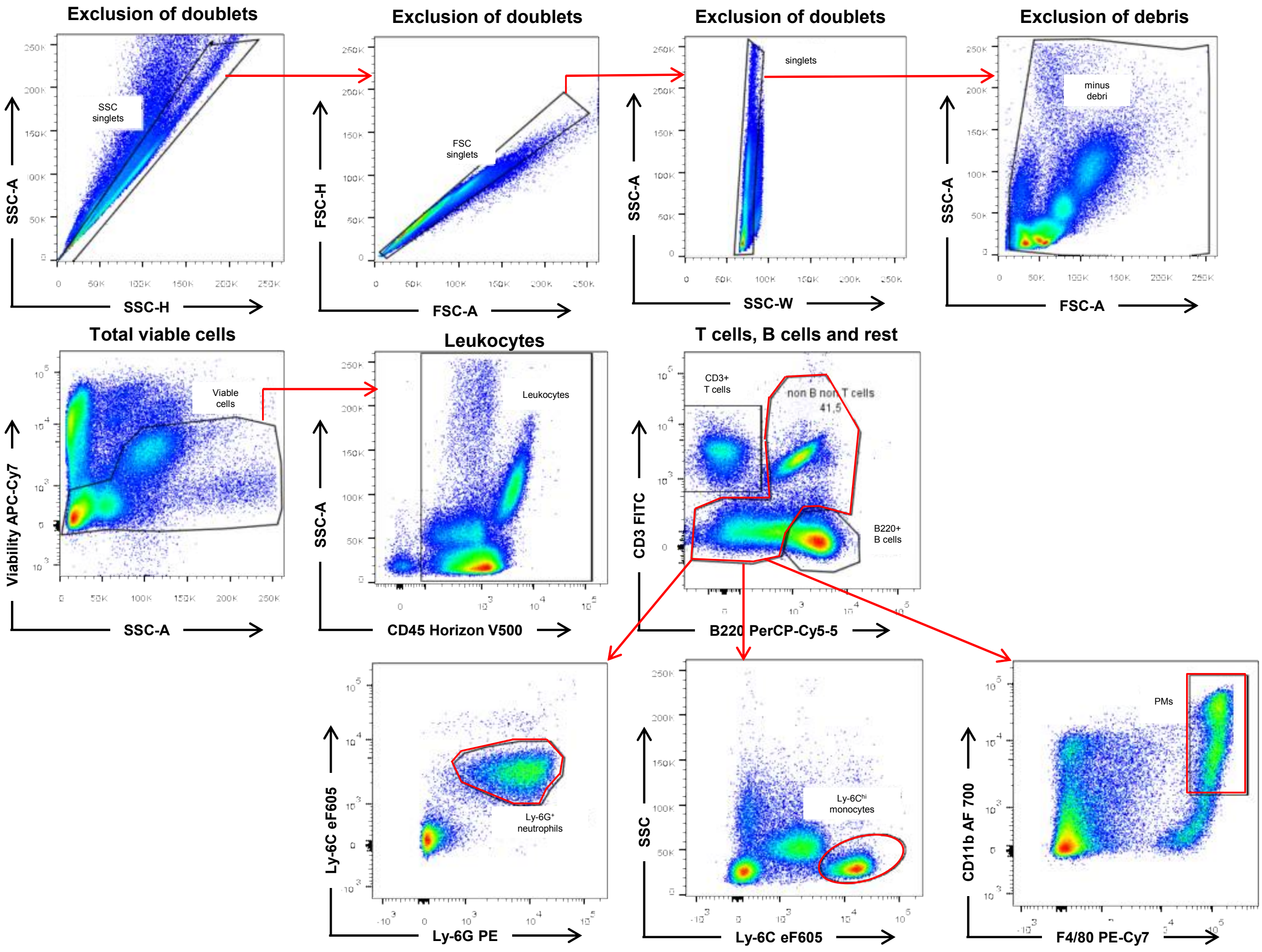

Exclusion of doublets
SSC singlets
SSC-A
SSC-H
Exclusion of doublets
FSC singlets
FSC-H
FSC-A
Exclusion of doublets
singlets
SSC-A
SSC-W
Exclusion of debris
minus debri
SSC-A
FSC-A
Total viable cells
Viable cells
Viability APC-Cy7
SSC-A
Leukocytes
Leukocytes
SSC-A
CD45 Horizon V500
T cells, B cells and rest
CD3+ T cells
non B non T cells
41,5
B220+ B cells
CD3 FITC
B220 PerCP-Cy5-5
Ly-6G+ neutrophils
Ly-6C eF605
Ly-6G PE
Ly-6Chi monocytes
SSC
Ly-6C eF605
PMs
CD11b AF 700
F4/80 PE-Cy7

Figure S7.

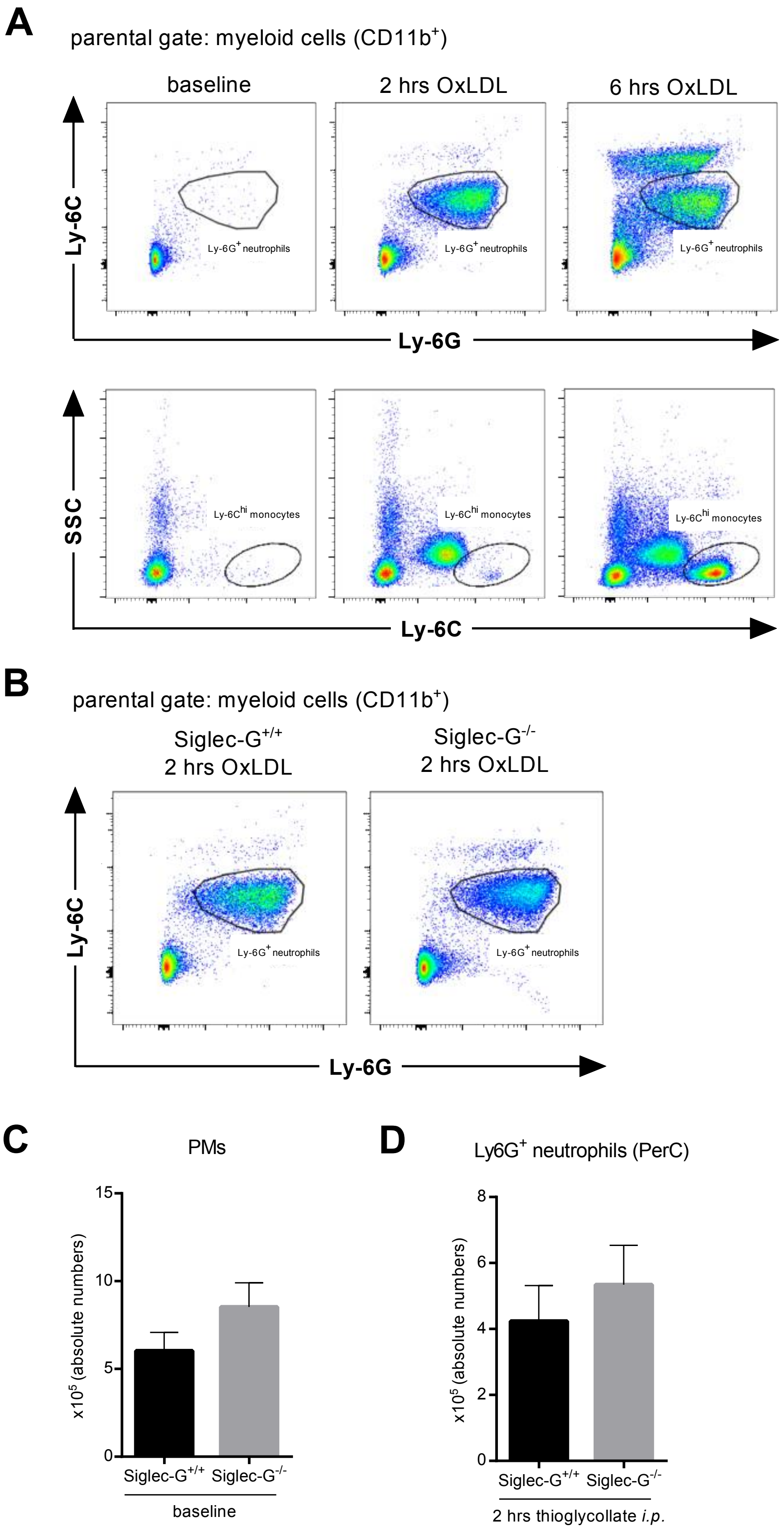

A
parental gate: myeloid cells (CD11b+)
baseline
2 hrs OxLDL
6 hrs OxLDL
Ly-6C
Ly-6G
Ly-6G+ neutrophils
SSC
Ly-6C
Ly-6Chi monocytes
B
parental gate: myeloid cells (CD11b+)
Siglec-G+/+
2 hrs OxLDL
Siglec-G-/-
2 hrs OxLDL
Ly-6C
Ly-6G
Ly-6G+ neutrophils
C
PMs
x10^5 (absolute numbers)
0
5
10
15
Siglec-G+/+
Siglec-G-/-
baseline
D
Ly6G+ neutrophils (PerC)
x10^5 (absolute numbers)
0
2
4
6
8
Siglec-G+/+
Siglec-G-/-
2 hrs thioglycollate i.p.

SUPPLEMENTAL FIGURE LEGENDS

Figure S1. Characterization of natural IgM responses in C57BL/6 Siglec-G-deficient mice, Related to Figure 1. (A) Absolute numbers of $B220^+IgM^+CD43^+$ B-1 cells from the spleen. (B) Absolute numbers of $B220^+IgM^+CD43^+CD5^+$ B-1a cells from the spleen. (C) Absolute numbers of $B220^+CD138^+$ plasmablasts from the spleen. (D) Absolute numbers of $B220^{lo}CD138^+$ plasma cells from the spleen. (E) Absolute numbers of $B220^+CD138^+$ plasmablasts from the bone marrow. (F) Absolute numbers of $B220^{lo}CD138^+$ plasma cells from the bone marrow. (G) IgM antibody binding to α-1,3-dextran, MDA-LDL, and CuOx-LDL in plasma was determined by ELISA. Plasma was diluted 1:100 and 1:500 and antibody binding was measured in triplicates. Shown are data of *Siglec-G*$^{+/+}$ *and Siglec-G*$^{-/-}$ mice at 8-12 weeks of age. Symbols represent individual mice. Horizontal bars represent the mean and error bars represent SEM of each group. * $P \leq 0.05$, ** $P \leq 0.01$, *** $P \leq 0.001$.

Figure S2. Immunological characterization of *Ldlr*$^{-/-}$ and *Ldlr*$^{-/-}$*Siglec-G*$^{-/-}$ after 8 weeks of atherogenic diet feeding, Related to Figure 1. (A) Absolute numbers of splenic $CD19^+IgM^{hi}CD43^+CD5^+CD138^+$ $CD93^+MHCII^+$ innate response activator (IRA) B cells. (B and C) Relative numbers of peritoneal cavity $B220^+CD5^-CD11b^+$ B-1b cells (B) and $B220^+CD5^-CD11b^-$ B2 cells (C) out of total B cells. (D) Relative numbers of peripheral blood $B220^+$ B cells. (E) Relative numbers of peripheral blood $Ly6C^+$ monocytes. (F) Relative numbers of peripheral blood $Ly6C^+$ inflammatory and $Ly6C^{lo}$ resident monocytes. (G-H) Titers of MDA-LDL IgG (G) and CuOx-LDL IgG (H) in plasma of *Ldlr*$^{-/-}$ and *Ldlr*$^{-/-}$*Siglec-G*$^{-/-}$ mice were determined by ELISA at baseline and after 8 weeks of atherogenic diet. Plasma samples were diluted 1:100 and 1:500 and antibody binding was measured in triplicates. Symbols represent individual mice (14-16 mice per group). Horizontal bars represent the mean and error bars represent SEM of each group. * $P \leq 0.05$, ** $P \leq 0.01$, *** $P \leq 0.001$.

Figure S3. Successful bone marrow reconstitution in *Ldlr*$^{-/-}$ recipient mice, Related to Figure 3. *Ldlr*$^{-/-}$ mice were reconstituted with bone marrow cells isolated from either *Siglec-G*$^{+/+}$ mice or *Siglec-G*$^{-/-}$ [KO] mice or a mixture of 80% bone marrow from *µMT* and 20% either *Siglec-G*$^{+/+}$ [WT] or *Siglec-G*$^{-/-}$ [KO] fed an atherogenic diet for 10 weeks. (A) At time of sacrifice bone marrow cells were collected from mice reconstituted with *Siglec-G*$^{+/+}$ [WT] and *Siglec-G*$^{-/-}$ [KO] and genomic DNA was extracted and amplified for Ldlr and Siglec-G genes. (For some mice DNA could not be recovered successfully). Tail DNA from *Siglec-G*$^{+/+}$, *Siglec-G*$^{-/-}$ and *Ldlr*$^{-/-}$ mice were used as positive and negative controls. (B) Validation of B cell restricted Siglec-G deficiency in *Ldlr*$^{-/-}$ mice after reconstitution with µMT+WT and µMT+*Siglec-G*$^{-/-}$ [KO] bone marrow after 10 weeks of atherogenic diet. DNA of sorted splenic B ($B220^+$) and non-B cells ($B220^-$) was isolated and pooled samples were analyzed by PCR for the Siglec-G gene. (C) Flow cytometry for Siglec-G expression. Representative histogram of Siglec-G expression on

B220$^+$ B cells in the spleens of mice reconstituted with µMT+WT and µMT+KO bone marrow after 10 weeks of atherogenic diet.

Figure S4. Immunological characterization of bone marrow chimeric *Ldlr*$^{-/-}$ mice after 10 weeks of atherogenic diet, Related to Figure 3. (A) Relative numbers of peripheral blood Ly6C$^+$ monocytes. (B) Relative numbers of peripheral blood Ly6C$^+$ inflammatory and Ly6C^{lo} resident monocytes. (C) Relative numbers of peripheral blood B220$^+$ B cells. (D) Relative numbers of B220$^+$CD5$^+$CD11b$^+$ B-1a cells out of total B cells from the peritoneal cavities. (E) Quantification of total IgG antibodies in plasma. Plasma samples were diluted 1:70,000 and measured in triplicates. (F-G) Binding of IgG antibodies to (F) MDA-LDL and (G) CuOx-LDL was determined by ELISA. Plasma samples were diluted between 1:100 and 1:500 and antibody binding was measured in triplicates. Data are expressed as RLU per 100 ms. Symbols represent individual mice. (H) IgM antibody repertoire to model epitopes of OxLDL. Titers of α-1,3-dextran IgM, MDA-LDL IgM and CuOx-LDL IgM in plasma were determined by ELISA. Plasma was diluted 1:100 and 1:500 and antibody binding was measured in triplicates. The data is plotted as the ratio of antigen-specific IgM to total IgM. Shown are data of *Ldlr*$^{-/-}$ mice reconstituted with *Siglec-G*$^{+/+}$ (n = 10) vs. *Siglec-G*$^{-/-}$ [KO] (n = 11) and µMT+*Siglec-G*$^{+/+}$ (n = 13) vs. µMT+*Siglec-G*$^{-/-}$ [KO] (n = 13) bone marrow after 10 weeks of atherogenic diet. Horizontal bars represent the mean and error bars represent SEM of each group. * $P \leq 0.05$, ** $P \leq 0.01$, *** $P \leq 0.001$.

Figure S5. Selective Siglec-G deficiency in B cells protects from atherogenic diet-induced hepatic and systemic inflammation, Related to Figure 4. (A, B) Quantification of infiltrating neutrophils (A) and macrophages (B) in liver sections of *Ldlr*$^{-/-}$ mice reconstituted with *Siglec-G*$^{+/+}$ [WT] vs. *Siglec-G*$^{-/-}$ [KO] and µMT+*Siglec-G*$^{+/+}$ [WT] vs. µMT+*Siglec-G*$^{-/-}$ [KO] and fed with an atherogenic diet for 10 weeks. Sections were stained with anti-Ly6G and anti-mac-1 antibody, and positively stained cells were counted and expressed as number of positive cells per mm^2. (C) Relative gene expression of *Icam, Vcam, Tnf-α, Il-18, Ccl5, Cxcl1, Cxcl2* and *Saa1* mRNA in livers of *Ldlr*$^{-/-}$ mice reconstituted with µMT+*Siglec-G*$^{+/+}$ [WT] vs. µMT+*Siglec-G*$^{-/-}$ [KO] bone marrow and fed an atherogenic diet for 10 weeks. The expression of individual genes was normalized to the house-keeping gene S12 and expressed relatively to the expression in µMT+*Siglec-G*$^{+/+}$ [WT] control mice. (D) Quantification of SAA levels in plasma of *Ldlr*$^{-/-}$ mice reconstituted with µMT+*Siglec-G*$^{+/+}$ [WT] vs. µMT+*Siglec-G*$^{-/-}$ [KO] bone marrow and fed an atherogenic diet for 10 weeks. Plasma samples were diluted 1:300 and measured in triplicates. (E) Quantification of a panel of chemokines and cytokines in plasma of *Ldlr*$^{-/-}$ mice reconstituted with µMT+*Siglec-G*$^{+/+}$ [WT] vs. µMT+*Siglec-G*$^{-/-}$ [KO] bone marrow and fed an atherogenic diet for 10 weeks by multiplex assay. Plasma samples were diluted 1:2. Data represent mean ± SEM of 9-13 mice per group. * $P \leq 0.05$, ** $P \leq 0.01$, *** $P \leq 0.001$.

Figure S6. Representative flow cytometry plots and gating strategy for analysis of peritoneal cell populations upon sterile peritonitis induction, Related to Figure 5. *Siglec-G*$^{+/+}$ and *Siglec-G*$^{-/-}$ mice were injected *i.p.* with OxLDL (25 µg/g body weight) and peritoneal exudate cells were harvested to monitor infiltration of immune cells. Representative flow cytometry plots and gating strategy is shown: After exclusion of doublets and cell debris by FSC and SSC, viable cells were identified by staining with a viability dye. CD45 was used to separate individual cell populations. T cells and B cells were identified by CD3 and B220 markers, respectively. The "non T and non B cells" gate ($CD3^-B220^-$ cells) was further used to define Ly-6G$^+$ neutrophils, Ly-6C^{hi} monocytes and CD11b$^+$F4/80$^+$ macrophages.

Figure S7. Characterization of sterile peritonitis in Siglec-G-deficient mice, Related to Figure 5. (A) Representative flow cytometry plots for Ly6G$^+$ neutrophils and Ly6G^{hi} inflammatory monocytes of C57BL/6 mice are shown at baseline, as well as 2, 6, and 12 hours after OxLDL injection. (B) Representative flow cytometry plots for Ly6G$^+$ neutrophils in the PLF of *Siglec-G*$^{+/+}$ vs. *Siglec-G*$^{-/-}$ mice 2 hours after *i.p.* OxLDL injection are shown. (C) Absolute numbers of CD11b$^+$F4/80$^+$ peritoneal macrophages (PMs) in the peritoneal lavage fluid at baseline in *Siglec-G*$^{+/+}$ and *Siglec-G*$^{-/-}$ mice. (D) Absolute numbers of Ly6G$^+$ neutrophils in the peritoneal lavage fluid after 2 hours of thioglycollate injection (25 µl/g body weight, *i.p.*) in *Siglec-G*$^{+/+}$ and *Siglec-G*$^{-/-}$ mice. Data represent mean and error bars represent SEM of 3-7 mice per group. * $P \leq 0.05$, ** $P \leq 0.01$, *** $P \leq 0.001$.

SUPPLEMENTAL EXPERIMENTAL PROCEDURES

Bone marrow transplantation

Bone marrow transplantation studies were performed as previously described (Binder et al., 2003, Fillatreau et al., 2002, Sage et al., 2012). Ten weeks-old male *Ldlr*$^{-/-}$ mice received a single dose of 9.5 Gy lethal irradiation and were subsequently injected intravenously via the retro-orbital plexus with 1×10^7 bone marrow cells from either *Siglec-G*$^{-/-}$ ($n = 12$) or *Siglec-G*$^{+/+}$ ($n = 12$) mice. In a separate experiment in order to assess the effects of selective deficiency of Siglec-G on B cells, irradiated *Ldlr*$^{-/-}$ mice were reconstituted with a mixture of 80% bone marrow from *µMT* mice (no B cells due to disruption of the membrane exon of the mu heavy chain gene) and 20% bone marrow from either *Siglec-G*$^{+/+}$ (n = 13) or *Siglec-G*$^{-/-}$ (n = 13) mice. After 4 weeks of recovery, mice were fed an atherogenic diet containing 21% milk fat and 0.2% cholesterol (Ssniff Spezialdiäten GmbH, Soest, Germany) for 10 weeks to induce lesion formation. Three mice (two in the *Siglec-G*$^{+/+}$ and one in *Siglec-G*$^{-/-}$) died during the study.

Evaluation of atherosclerosis

The extent of atherosclerosis was assessed in the entire aorta and in cross-sections of the aortic origin, as previously described (Cardilo-Reis et al., 2012). Histological sections of the paraffin-embedded aortic origin were stained with

a modified elastic-trichrome stain for quantification of lesion size and size of necrotic areas. Macrophage areas were assessed by immunohistochemistry using anti-mouse-mac-3 antibodies (rat anti-mouse, clone M3/84, BD-Biosciences Pharmingen, San Diego, California, USA). For the quantification of lesional collagen content, sections were stained with Sirius Red (Direct Red 80, Sigma Aldrich). For the presence of IgM, immunohistochemistry was performed using an antibody against mouse IgM, µ-chain specific (Sigma-Aldrich). For indicated experimental studies, innominate arteries were isolated and embedded in paraffin as described (Reardon et al., 2003). All photographed images were quantified using Adobe Photoshop CS5 and analyzed using ImageJ 1.47 software.

Flow cytometry

At time of sacrifice, peritoneal exudates cells (PECs) were collected by lavaging the peritoneum with 10 ml of sterile HBSS + 2% FBS. PECs were pelleted; cells were counted and subsequently processed for flow cytometry. Spleens and bone marrow were harvested and passed through a cell strainer to obtain a single cell suspension and red blood cells were lysed using a commercial lysis buffer (Morphisto). Total white blood cells were isolated from blood collected in EDTA-tubes via the vena cava. Blood was diluted 1:1 in a solution of PBS containing 2% Dextran (Sigma-Aldrich) and incubated for 40 min at 37ºC to separate the red blood cells. The supernatant was harvested and used for flow cytometry analysis. 1×10^6 cells were used for flow cytometry staining, and 5×10^6 cells were lysed in 350 µl RLT RNA lysis buffer (Qiagen) and stored at -20°C.

For flow cytometric analyses, cells were blocked with anti-mouse CD16/32 blocking Ab (anti-mouse CD16/CD32, clone 93, 0.5 µg/0.5×10^6 cells, eBioscience) for 20 min at 4°C and stained for 30 min with following antibodies: PerCP-Cy5.5-labeled anti-CD45R (B220) (clone RA3-6B2, 1:800, eBioscience), FITC-labeled anti-CD23 (clone B3B4, 1:600, BD Biosciences – Pharmingen), APC-labeled anti-IgM (clone II/41, 1:600, eBioscience), phycoerythrin (PE)-labeled anti-CD43 (clone S7, 1:600, BD Biosciences – Pharmingen), biotin-labeled anti-CD21/CD35 (CR2/CR1) (clone 7E9, 1:200, BioLegend), PE-labeled anti-CD3e (clone 145-2C11, 1:400, eBioscience), FITC-labeled anti-CD4 (clone GK1.5, 1:400, eBioscience), APC-labeled anti-CD8a (clone 53-6.7, 1:400, eBioscience), FITC-labeled anti-kappa (clone 197.1, 1:400, BD Biosciences – Pharmingen), Biotin-labeled anti-lambda (clone RML-42, 1:400, BioLegend), PE-labeled anti-IgD (clone 11-26c (11-26), 1:200, eBioscience), PE-labeled anti-CD93 (clone AA4.1, 1:400, eBioscience), APC-labeled anti-Siglec-G (clone SH2.1, 1:400, eBioscience), APC-labeled anti-CD11b/Mac-1 (clone M1/70, 1:600, eBioscience), FITC-labeled anti CD11b (clone M1/70, 1:600, eBioscience), PE-labeled anti-CD5 (clone 53-7.3, 1:100, eBioscience), FITC-labeled anti-Ly6C (clone HK14, 1:200, BioLegend), PE-labeled anti-Ly6G (clone 1A8, 1:2000, BioLegend), V500-labeled anti-CD45 (clone 30-F11, 1:200, BD Biosciences – Pharmingen), BV570-labeled anti-Ly-6C (clone HK1.4, 1:100, BioLegend), eFluor 780-labeled fixable viability dye (1:1000, eBioscience), AF700-labeled anti-CD11b (clone M1/70, 1:700, eBioscience), Brilliant Violet 570-labelled anti-CD19 (1:100, clone 6D5, BioLegend), PE-labelled anti-GM-CSF (1:100, clone MP1-22E9, BioLegend), PECy7-labelled anti CD93 (1:100, clone AA4.1, eBioscience), biotin-labeled anti-CD5 (Ly-1) (1:200, clone 53-7.3, BD Pharmingen), AF700-labelled anti-MHCII (1:100, clone M5/114.15.2, eBioscience), FITC-labeled anti-IgD (1:800, clone 11-26c, eBioscience), biotin-labeled anti-CD138 (Syndecan 1) (1:200, clone 281-2, BioLegend). All stains were performed in 100 µl of FACS buffer (PBS + 10% FCS) for 30 min

at 4°C in darkness, followed by two washing steps. Stained cell populations were analyzed by multiparameter flow cytometry using a BD FACSCalibur (BD Bioscience, Franklin Lakes, New Jersey, USA) or BD FACS Fortessa, respectively. Either 1×10^5 or 1×10^6 cells per sample were stained and acquired. Dead cells and doublets were excluded by forward- and side-scatter and data were analyzed using the FlowJo version 10 data analysis software (Tree Star Inc., Oregon Corporation, Ashland, USA).

Clinical chemistry and lipid analyses

At time of sacrifice, blood was collected via the vena cava into EDTA tubes (MiniCollect® 1 ml K_3EDTA Blood Collection Tube, Greiner Bio-One), centrifuged for 30 min at 1000 rpm, and aliquots of plasma were stored at -80°C for further analyses. Total plasma cholesterol and triglycerides were measured by enzymatic methods using an automated analyzer AU5400 – Chemistry System (Beckman Coulter, Brea, California, USA). Liver lipid levels were measured by enzymatic methods using the CHOD-PAP assay (Roche Diagnostics, Basel, Switzerland) for cholesterol and a triglyceride determination kit (Sigma-Aldrich). Non-esterified fatty acids (NEFA) in the liver were determined using the NEFA-C kit (ACS-ACOD method; Wako Chemicals, Neuss, Germany). All measurements were done according to the manufacturer's protocols using a Benchmark 550 Micro-plate Reader (Bio-Rad, Hercules, CA). Protein content was measured with the bicinchoninic acid (BCA) method (Pierce, Rockford, IL) according to manufacturer’s protocol. Data are expressed as µg lipid per µg protein.

ALT levels were measured in plasma (30 µl, 1:3 diluted in physiological NaCl) of each individual mouse by use of the Reflotron system in combination with the strips for alanine aminotransferase (Roche Diagnostics, Almere, The Netherlands) according to the manufacturer's instructions.

Gene expression analysis

Liver. Total RNA was isolated from mouse liver tissue and was reversely transcribed using the iScript cDNA synthesis kit (Bio-Rad, Hercules, USA). Quantitative real-time PCR was performed using SensiMix™ SYBR green with ROX dye (Bioline**,** Randolph, USA) on an ABI7900HT instrument (Applied Biosystems). The relative expression level of target genes was determined with the LinRegPCR software (http://www.hfrc.nl, (Ruijter et al., 2009), and obtained values were normalized to the housekeeping gene ribosomal protein S12 (Rps12). Values are expressed as the relative expression compared to the control group.

Primer sequences:

Cxcl2-forward: 5’-AGTGAACTGCGCTGTCAATGC-3’;

Cxcl2-reverse: 5’-AGGCAAACTTTTTGACCGCC-3’;

TNF-α-forward: 5’-CATCTTCTCAAAATTCGAGTGACAA-3’;

TNF-α-reverse: 5’-TGGGAGTAGACAAGGTACAACCC-3’;

Cxcl1-forward: 5’-TGCACCCAAACCGAAGTCAT-3’;

Cxcl1-reverse: 5’-TTGTCAGAAGCCAGCGTTCAC-3’;

SAA1-forward: 5’-GGCTGCTGAGAAAATCAGTGATG-3’;

SAA1-reverse: 5’-TCAGCAATGGTGTCCTCATGTC-3’;

VCAM-forward: 5’-GTGTTGAGCTCTGTGGGTTTTG-3’;

VCAM-reverse: 5’-TTAATTACTGGATCTTCAGGGAATGAG-3’;

ICAM-forward: 5’-CTACCATCACCGTGTATTCGTTTC-3’;

ICAM-reverse: 5’-CGGTGCTCCACCATCCA-3’;

IL-18-forward: 5’-ACAACTTTGGCCGACTTCAC-3’;

IL-18-reverse: 5’-GGGTTCACTGGCACTTTGAT-3’;

CCL5-forward: 5’-GGAGTATTTCTACACCAGCAGCAA-3’;

CCL5-reverse: 5’-GCGGTTCCTTCGAGTGACA-3’;

S12-forward: 5’-GGAAGGCATAGCTGCTGGAGGTGT-3’;

S12-reverse: 5’-CCTTCGATGACATCCTTGGCCTGAG-3’.

Validation of successful BMT

At time of sacrifice, bone marrow cells from all mice were collected and genomic DNA was extracted by incubating cells in 40 µl of lysis buffer (1 M Tris/HCl, pH 8.0, 500 mM EDTA, 10% SDS, 2 M NaCl, 0.4 mg/ml Proteinase K) at 55°C overnight with gentle mixing, followed by 10 min incubation at 99°C to inactivate Proteinase K. The purified genomic DNA was diluted 1:10 in H_2O and amplified by PCR for the Ldlr and Siglec-G gene to identify successful bone marrow reconstitution with donor bone marrow. In order to assess selective Siglec-G deficiency on B cells, B cells and non B cells of splenocytes from recipient mice were separated using anti-CD45R (B220) MicroBeads (Milteny Biotec) according to the manufacturer’s protocol. Collected cell fractions were lysed in RLT Plus buffer (Qiagen) and stored at -20°C. Genomic DNA was purified with the AllPrep DNA/RNA Micro Kit (Qiagen) according to the manufacturer’s protocol and equal amounts (50 ng) were used for PCR to amplify the genes of interest.

Primer sequences:

Siglec-G-forward: 5'-CCGCTCGAGATGTTGTCCCGCGGGTGGTTTCA-3';

Siglec-G-reverse: 5'-CCGGAATTCCTTCCTCTTGAGAGAACCTTTGTTC-3';

Ldlr-forward: 5'-ACCCCAAGACGTGCTCCCAGGATG-3';

Ldlr-reverse: 5'-CGCAGTGCTCCTCATCTGACTTGT-3';

neo cassette primer: 5'-AGGTGAGATGACAGGAGA-3'.

SUPPLEMENTAL REFERENCES

BINDER, C. J., HORKKO, S., DEWAN, A., CHANG, M. K., KIEU, E. P., GOODYEAR, C. S., SHAW, P. X., PALINSKI, W., WITZTUM, J. L. & SILVERMAN, G. J. 2003. Pneumococcal vaccination decreases atherosclerotic lesion formation: molecular mimicry between Streptococcus pneumoniae and oxidized LDL. *Nature medicine,* 9**,** 736-43.

CARDILO-REIS, L., GRUBER, S., SCHREIER, S. M., DRECHSLER, M., PAPAC-MILICEVIC, N., WEBER, C., WAGNER, O., STANGL, H., SOEHNLEIN, O. & BINDER, C. J. 2012. Interleukin-13 protects from atherosclerosis and modulates plaque composition by skewing the macrophage phenotype. *EMBO molecular medicine,* 4**,** 1072-86.

FILLATREAU, S., SWEENIE, C. H., MCGEACHY, M. J., GRAY, D. & ANDERTON, S. M. 2002. B cells regulate autoimmunity by provision of IL-10. *Nature immunology,* 3**,** 944-50.

REARDON, C. A., BLACHOWICZ, L., LUKENS, J., NISSENBAUM, M. & GETZ, G. S. 2003. Genetic background selectively influences innominate artery atherosclerosis: immune system deficiency as a probe. *Arteriosclerosis, thrombosis, and vascular biology,* 23**,** 1449-54.

RUIJTER, J. M., RAMAKERS, C., HOOGAARS, W. M., KARLEN, Y., BAKKER, O., VAN DEN HOFF, M. J. & MOORMAN, A. F. 2009. Amplification efficiency: linking baseline and bias in the analysis of quantitative PCR data. *Nucleic acids research,* 37**,** e45.

SAGE, A. P., TSIANTOULAS, D., BAKER, L., HARRISON, J., MASTERS, L., MURPHY, D., LOINARD, C., BINDER, C. J. & MALLAT, Z. 2012. BAFF receptor deficiency reduces the development of atherosclerosis in mice--brief report. *Arteriosclerosis, thrombosis, and vascular biology,* 32**,** 1573-6.
