# Supplementary material for: Sialic Acid-Binding Immunoglobulin-like Lectin G Promotes Atherosclerosis and Liver Inflammation by Suppressing the Protective Functions of B-1 Cells
Source: Cell Rep. 2016 Mar 3;14(10):2348–61. doi: 10.1016/j.celrep.2016.02.027 (PMC4802221; doi:10.1016/j.celrep.2016.02.027)
Supplement: Document S2. Article plus Supplemental Information [file mmc2.pdf]

Cell Reports

Article

Sialic Acid-Binding Immunoglobulin-like Lectin G Promotes Atherosclerosis and Liver Inflammation by Suppressing the Protective Functions of B-1 Cells

Graphical Abstract

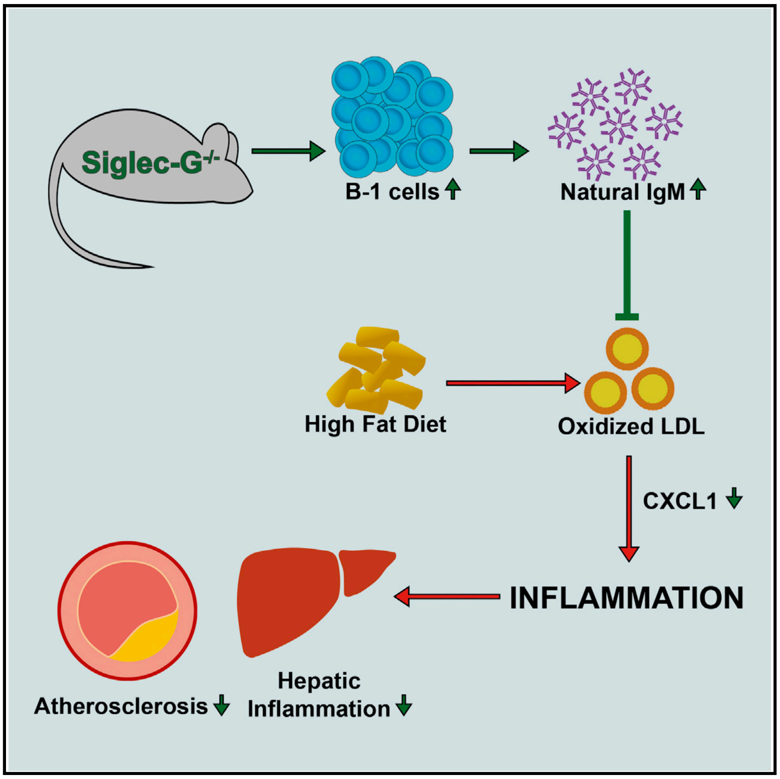

Highlights

- Siglec-G deficiency reduces atherosclerosis and hepatic inflammation in Ldlr KO mice
- B cells secreting natural IgM mediate the protective effects of Siglec-G deficiency
- Siglec-G deficiency protects from inflammation induced by oxidized LDL
- CXCL1 is a common mediator of inflammation that is decreased by Siglec-G deficiency

Authors

Sabrina Gruber, Tim Hendrikx, Dimitrios Tsiantoulas, ..., Ronit Shiri-Sverdlov, Lars Nitschke, Christoph J. Binder

Correspondence

christoph.binder@meduniwien.ac.at

In Brief

Gruber et al. demonstrate that Siglec-G deficiency protects from oxidized LDL-induced inflammation through the expansion of B-1 cells secreting natural IgM antibodies, leading to reduced atherosclerosis and hepatic inflammation. CXCL1 represents a common pro-inflammatory factor that is reduced as a result of Siglec-G deficiency.

Gruber et al., 2016, Cell Reports *14*, 2348–2361
March 15, 2016 ©2016 The Authors
http://dx.doi.org/10.1016/j.celrep.2016.02.027

OPEN ACCESS
CellPress

Cell Reports
Article

Sialic Acid-Binding Immunoglobulin-like Lectin G Promotes Atherosclerosis and Liver Inflammation by Suppressing the Protective Functions of B-1 Cells

Sabrina Gruber,[1,2] Tim Hendrikx,[1,2,3] Dimitrios Tsiantoulas,[1,2] Maria Ozsvar-Kozma,[1,2] Laura Göderle,[1,2] Ziad Mallat,[4] Joseph L. Witztum,[5] Ronit Shiri-Sverdlov,[3] Lars Nitschke,[6] and Christoph J. Binder[1,2,*]
[1]CeMM Research Center for Molecular Medicine of the Austrian Academy of Sciences, 1090 Vienna, Austria
[2]Department of Laboratory Medicine, Medical University of Vienna, 1090 Vienna, Austria
[3]Department of Molecular Genetics, School of Nutrition and Translational Research in Metabolism (NUTRIM), Maastricht University, 6229 ER Maastricht, the Netherlands
[4]Division of Cardiovascular Medicine, Department of Medicine, University of Cambridge, CB2 0SZ Cambridge, UK
[5]Division of Endocrinology and Metabolism, Department of Medicine, University of California San Diego, La Jolla, CA 92110, USA
[6]Division of Genetics, Department of Biology, University of Erlangen-Nuremberg, 91058 Erlangen, Germany
*Correspondence: christoph.binder@meduniwien.ac.at
http://dx.doi.org/10.1016/j.celrep.2016.02.027
This is an open access article under the CC BY license (http://creativecommons.org/licenses/by/4.0/).

SUMMARY

Atherosclerosis is initiated and sustained by hypercholesterolemia, which results in the generation of oxidized LDL (OxLDL) and other metabolic byproducts that trigger inflammation. Specific immune responses have been shown to modulate the inflammatory response during atherogenesis. The sialic acid-binding immunoglobulin-like lectin G (Siglec-G) is a negative regulator of the functions of several immune cells, including myeloid cells and B-1 cells. Here, we show that deficiency of Siglec-G in atherosclerosis-prone mice inhibits plaque formation and diet-induced hepatic inflammation. We further demonstrate that selective deficiency of Siglec-G in B cells alone is sufficient to mediate these effects. Levels of B-1 cell-derived natural IgM with specificity for OxLDL were significantly increased in the plasma and peritoneal cavity of Siglec-G-deficient mice. Consistent with the neutralizing functions of OxLDL-specific IgM, Siglec-G-deficient mice were protected from OxLDL-induced sterile inflammation. Thus, Siglec-G promotes atherosclerosis and hepatic inflammation by suppressing protective anti-inflammatory effector functions of B cells.

INTRODUCTION

Atherosclerosis is a lipid-driven chronic disease of the artery wall and the underlying cause of heart attacks and strokes, which accounts for the majority of mortalities and morbidities in the world (Libby et al., 2011). It is characterized by chronic inflammatory responses to endogenous sterile triggers, such as oxidized LDL (OxLDL), dying cells, and their metabolic byproducts that trigger tissue inflammation if not efficiently cleared (Tabas, 2010; Hotamisligil, 2006). Persistence of this inflammatory response or its impaired resolution paves the way for chronic inflammatory responses, which have been shown to propagate associated pathologies such as vascular and hepatic inflammation (Tall and Yvan-Charvet, 2015). Thus, there is growing interest in identifying mechanisms that enhance the immune system's capacity to prevent endogenously triggered inflammation and/or promote its resolution.

B cells, which can be subdivided into B-1 and B2 cells, are emerging players in the chronic inflammation of metabolic diseases, such as obesity, diabetes, and atherosclerosis (Tsiantoulas et al., 2014; Winer et al., 2014; Zouggari et al., 2013; Perry et al., 2012). B2 cells, which include follicular (FO) B cells and marginal zone (MZ) B cells, have been shown to promote atherosclerotic lesion formation in murine models of atherosclerosis via mechanisms that are largely unclear (Kyaw et al., 2010; Ait-Oufella et al., 2010). On the other hand, selective transfer of B-1 cells, which can be further divided into B-1a and B-1b cells, protects mice from atherosclerosis (Kyaw et al., 2011; Rosenfeld et al., 2015). One of the main functions of B-1 cells is the production of natural IgM antibodies (NAb), which are pre-existing germline encoded antibodies that arise without any conventional T cell help and comprise approximately 80% of IgM antibodies in unchallenged mice (Baumgarth et al., 2005). B-1a cells seem to exhibit their atheroprotective effects via the secretion of NAb (Tsiantoulas et al., 2014). Indeed, atherosclerosis-prone soluble IgM-deficient mice develop accelerated atherosclerosis, though the exact mechanism by which NAb protect is not entirely clear (Lewis et al., 2009). We and others have suggested that NAb promote the neutralization and clearance of self-antigens, such as dying cells and oxidized lipids (Tsiantoulas et al., 2012). These studies indicate the importance of selective regulation of individual B cell subsets for appropriate responses to inflammatory triggers. Moreover, the role of B-1 cells in atherosclerosis has only been studied in immune-compromised animals, and their role in animals that do not lack major compartments of the immune

CrossMark

system remains elusive. In this regard, the sialic acid-binding immunoglobulin-like lectin G (Siglec-G) is of particular interest as it acts as negative regulator of the B-1a cell population size, presumably via inhibiting B cell receptor dependent signaling (Hoffmann et al., 2007; Ding et al., 2007). We and others have previously shown that mice deficient in Siglec-G exhibit a nearly 10-fold expansion of B-1a cells along with a robust increase in total serum IgM (Hoffmann et al., 2007; Ding et al., 2007). Moreover, we also found that Siglec-G deficiency results in an expansion of IgM with specificity for oxidation-specific epitopes (OSE), which represent prototypic metabolic byproducts present on OxLDL, dying cells, and circulating microparticles (Chou et al., 2009; Tsiantoulas et al., 2015; Chang et al., 1999, 2004; Jellusova et al., 2010). As excessive accumulation of OSE has been suggested to be a key driver for inflammatory reactions in metabolic diseases, such as atherosclerosis, non-alcoholic steatohepatitis, and diabetes (Miller et al., 2011; Walenbergh et al., 2013; Horie et al., 1997), targeting Siglec-G may have beneficial therapeutic effects in chronic inflammation.

The expansion of B-1a cells has also been associated with increased autoimmunity (Chan et al., 1997; Pao et al., 2007; Ishida et al., 2006), which could accelerate atherosclerosis (Roman and Salmon, 2007; Ma et al., 2008). Siglec-G deficiency has been shown to result in an earlier onset of autoimmune disease in the Murphy Roths Large/lymphoproliferative (MRL/lpr) lupus mouse model and leads to mild autoimmunity in aging mice with an over-activation of adaptive T and B cells (Müller et al., 2015; Bökers et al., 2014). In addition, Siglec-G has also been found to be expressed in and influence responses of myeloid cells. For example, Siglec-G has been shown to be upregulated by RNA viruses and to inhibit retinoic acid-inducible gene 1 (RIG-I) mediated IFN-β secretion by macrophages and dendritic cells. In line with this, vesicular stomatitis virus (VSV)-infected Siglec-G-deficient mice were found to display increased IFN-β production and decreased viral load compared to control mice (Chen et al., 2013). Moreover, dendritic cells of Siglec-G-deficient mice have been found to exhibit increased pro-inflammatory cytokine secretion in response to multiple danger-associated molecular patterns (DAMPs) (e.g., HSP70, HSP90, and HMGB1). A detrimental role of Siglec-G deficiency is further supported by the findings that Siglec-G-deficient mice exhibit increased mortality in models of acetaminophen-induced liver necrosis (Chen et al., 2009) and cecal ligation and puncture-induced sepsis (Chen et al., 2011). Thus, all studies so far indicate that Siglec-G functions as negative regulator of inflammation, and Siglec-G deficiency may actually propagate inflammatory responses. Because pro-inflammatory cytokine production is a hallmark of metabolic inflammation, the role of Siglec-G and the consequences of Siglec-G deficiency in chronic inflammation and specifically in atherosclerosis are entirely unknown.

Here, we investigated the role of Siglec-G in sterile chronic inflammation in vivo. We demonstrate that total as well as B cell-specific Siglec-G deficiency reduces atherosclerotic lesion formation as well as hepatic inflammation in hypercholesterolemic *Ldlr*$^{-/-}$ mice. Moreover, we show that Siglec-G-deficient mice are protected form OxLDL-induced inflammation in vivo.

RESULTS

Siglec-G Deficiency in Cholesterol-Fed Ldlr$^{-/-}$ Mice Increases B-1a Cells and Natural IgM Antibodies

Siglec-G deficiency has been previously shown to result in an expansion of OSE-specific NAb, which are hypothesized to possess robust anti-inflammatory properties, particularly against products of increased oxidative stress (Jellusova et al., 2010). In agreement with previous data, non-atherosclerotic *Siglec-G*$^{-/-}$ mice have—compared to *Siglec-G*$^{+/+}$ control mice—significantly increased numbers of B-1a cells in the spleen, increased numbers of CD138^{+} plasmablasts (B220^{+}) and plasma cells (B220^{lo}) in the spleen and bone marrow, and increased levels of OSE-specific IgM antibodies in the plasma (Figures S1A–S1G). In order to investigate the effect of Siglec-G deficiency in atherosclerosis, *Ldlr*$^{-/-}$*Siglec-G*$^{-/-}$ mice as well as control *Ldlr*$^{-/-}$ mice were fed an atherogenic diet for 8 weeks. Siglec-G deficiency did not affect body weight, or plasma total cholesterol (TC), or triglycerides (TG) (Table 1). Moreover, there was no significant difference in frequencies of total or Ly6C^{hi} and Ly6C^{lo} monocytes in the peripheral blood of these mice (Figures S2E and S2F). Frequencies of peripheral blood B220^{+} B cells were also not different between the two groups (Figure S2D).

Consistent with the previously described effect of Siglec-G deficiency on B cells, numbers of splenic CD43^{+} B-1 cells were increased in *Ldlr*$^{-/-}$*Siglec-G*$^{-/-}$ mice (Figures 1A and 1B). Numbers of CD3^{+} T cells, CD23^{+} FO B cells, and MZB cells were not different between the groups (Table 1). Notably, the numbers of the recently identified pro-atherogenic subset of innate response activator (IRA) B cells in the spleen were significantly increased in *Ldlr*$^{-/-}$*Siglec-G*$^{-/-}$ mice compared to *Ldlr*$^{-/-}$ mice (Hilgendorf et al., 2014) (Figure S2A). Moreover, compared to *Ldlr*$^{-/-}$ mice, *Ldlr*$^{-/-}$*Siglec-G*$^{-/-}$ mice exhibited a significantly increased frequency of B-1a cells in the peritoneal cavity (Figures 1C and 1D), while the frequencies of B-1b or B2 cells were not significantly different between the groups (Figures S2B and S2C).

We then assessed both total and OSE-specific IgM and IgG antibody levels in plasma at baseline and after 8 weeks of atherogenic diet. Levels of IgM antibodies were significantly increased in *Ldlr*$^{-/-}$*Siglec-G*$^{-/-}$ mice at both time points (Figure 1E), while levels of total IgG antibodies were not different (Figure 1F). Moreover, IgM titers to MDA-LDL as well as to CuOx-LDL were also higher in *Ldlr*$^{-/-}$*Siglec-G*$^{-/-}$ mice at baseline and even further increased in response to atherogenic diet feeding compared to controls (Figures 1G and 1H). Importantly, expression of the antigen-specific IgM as a ratio of total IgM, revealed a preferential expansion of IgM to MDA-LDL and CuOx-LDL, while the relative levels of IgM to the atherosclerosis-irrelevant antigen α-1,3-dextran were decreased (Figure 1I). On the other hand, IgG titers to these antigens were elevated in *Ldlr*$^{-/-}$*Siglec-G*$^{-/-}$ mice only at baseline, but the differences disappeared after 8 weeks of atherogenic diet (Figures S2G and S2H). Thus, even in the presence of extreme hypercholesterolemia, Siglec-G deficiency results in an expansion of B-1a cells and a concomitant increase of total and OSE-specific IgM.

Table 1. Parameters Overview of Experimental Groups: Atherosclerosis Study

	Ldlr$^{-/-}$ (n = 16)	*Ldlr*$^{-/-}$*Siglec-G*$^{-/-}$ (n = 14)
Metabolic Parameters[a]		
Weights (g)	32.15 ± 0.53	31.2 ± 0.90
TC (mg/dL)	1,655.36 ± 70.24	1,633.65 ± 88.09
TG (mg/dL)	1,257.14 ± 55.37	1,284.38 ± 61.18
Atherosclerosis		
en face (% of aorta)	4.22 ± 0.25	2.22 ± 0.20***
Innominate (× 10^4 mm^2)	0.99 ± 0.17	0.43 ± 0.16*
Aortic origin (× 10^4 μm^2/section)	7.08 ± 0.88	8.69 ± 1.14
Necrotic area (% of total lesion area)	14.8 ± 4.0	4.65 ± 1.21*
mac-3^{+} (% of cellular area)	65.83 ± 3.78	51.61 ± 2.53**
Hepatic Inflammation		
Liver cholesterol (μg TC/μg protein)	0.19 ± 0.01	0.19 ± 0.02
Liver TG (μg TG/μg protein)	0.68 ± 0.04	0.62 ± 0.06
Liver free fatty acids (μg FFA/μg protein)	0.42 ± 0.02	0.37 ± 0.04
ALT (U/l)	72.9 ± 8.1	80.4 ± 6.6
Mac-1 (# positive cells/mm^2)	150.04 ± 6.74	118.58 ± 6.14**
Ly6G (# positive cells/mm^2)	68.38 ± 2.61	55.91 ± 2.32**
Serum Antibody Titers		
Total IgM (mg/ml)		
Baseline	0.26 ± 0.05	1.15 ± 0.05***
8 weeks HFD	0.87 ± 0.06	1.36 ± 0.06***
MDA-LDL IgM (RLU/100 ms)		
Baseline	30,735 ± 1,534	54,680 ± 2,101***
8 weeks HFD	54,378 ± 2,196	80,773 ± 2,467***
CuOx-LDL IgM (RLU/100 ms)		
Baseline	3,767 ± 585	12,459 ± 1,894***
8 weeks HFD	19,549 ± 2,443	40,649 ± 2,698***
Total IgG (mg/ml)		
Baseline	0.24 ± 0.02	0.39 ± 0.07
8 weeks HFD	0.72 ± 0.06	0.89 ± 0.07
MDA-LDL IgG (RLU/100 ms)		
Baseline	14,879 ± 1,855	22,392 ± 2,383*
8 weeks HFD	20,646 ± 1,800	23,699 ±1,870
CuOx-LDL IgG (RLU/100 ms)		
Baseline	1,473 ± 91	3,220 ± 525**
8 weeks HFD	3,267 ± 356	4,463 ± 449
Spleen (Absolute Numbers × 10^6)		
T cells ($CD3^{+}$)	34.18 ± 2.30	37.97 ± 3.04
B cells ($B220^{+}IgM^{+}$)	33.14 ± 2.44	38.64 ± 4.25
B-1 cells ($B220^{+}IgM^{+}CD43^{+}$)	2.63 ± 0.32	5.56 ± 0.73***
MZ B cells ($B220^{+}CD43^{-}CD23^{-}CD21^{hi}$)	0.71 ± 0.11	0.54 ± 0.09
T2/FO B cells ($B220^{+}CD43^{-}CD23^{+}CD21^{+}$)	26.87 ± 2.26	34.0 ± 3.71
T1 B cells ($B220^{+}CD43^{-}CD23^{-}CD21^{lo}$)	1.96 ± 0.18	2.71 ± 0.36
$B220^{+}CD43^{-}$ $CD23^{-}$ $CD21^{+}$	1.50 ± 0.15	1.84 ± 0.15
IRA B cells ($CD19^{+}IgM^{high}CD43^{+}CD5^{+}$ $CD138^{+}CD93^{+}MHCII^{+}$)	0.09 ± 0.01	0.18 ± 0.03**
Peritoneal Cavity (% Viable Cells)		
B-1a cells ($B220^{+}CD11b^{+}CD5^{+}$) out of B cells	27.74 ± 1.59	37.78 ± 2.60**
B-1b cells ($B220^{+}CD11b^{+}CD5^{-}$) out of B cells	15.57 ± 1.46	12.39 ± 1.41
B2 cells ($B220^{+}CD11b^{-}CD5^{-}$) out of B cells	35.29 ± 1.84	33.2 ± 1.84
T cells ($B220^{-}CD5^{+}$) out of total	8.99 ± 1.10	9.42 ± 0.86

See also Figure S5.
[a]Data are shown as mean ± SEM (* $p \leq 0.05$, ** $p \leq 0.01$, and *** $p \leq 0.001$).

Total and B Cell-Selective Siglec-G Deficiency Protects from Atherosclerosis

We then quantified the extent of atherosclerosis in the entire aorta of both groups of mice by *en face* analyses, which revealed a 50% reduced lesion size as a result of Siglec-G deficiency (Figure 2A). Decreased atherosclerosis was also found in the innominate arteries of *Ldlr*$^{-/-}$*Siglec-G*$^{-/-}$ mice compared to *Ldlr*$^{-/-}$ mice (Figure 2B). Moreover, while cross-sectional analyses of the aortic origin did not reveal differences in lesion size between the two groups (Figure 2C), lesions of *Ldlr*$^{-/-}$*Siglec-G*$^{-/-}$ mice were clearly less complex with smaller necrotic areas and reduced macrophage content (Figures 2D and 2E). Lesional collagen content was not significantly different between the two groups (Figure 2F), while deposition of IgM was significantly increased in lesions of *Ldlr*$^{-/-}$*Siglec-G*$^{-/-}$ mice (Figure 2G). Thus, Siglec-G deficiency results in a profound reduction of lesion size and complexity.

To identify whether the observed protective effects of Siglec-G deficiency are mainly conferred by B cells, we generated bone marrow chimeric *Ldlr*$^{-/-}$ mice with a selective Siglec-G deficiency on B cells using a previously established method (Sage et al., 2012; Fillatreau et al., 2002). *Ldlr*$^{-/-}$ mice were lethally irradiated and reconstituted with (1) bone marrow from *Siglec-G*$^{-/-}$ mice or *Siglec-G*$^{+/+}$ littermate controls to assess the role of Siglec-G deficiency in hematopoietic cells in general, and (2) a mixture of 80% bone marrow from B cell-deficient *μMT* mice and 20% from either *Siglec-G*$^{-/-}$ mice or *Siglec-G*$^{+/+}$ littermate controls to assess Siglec-G deficiency specifically in B cells. After a 4 week recovery period, mice were fed an atherogenic diet for 10 weeks. Successful

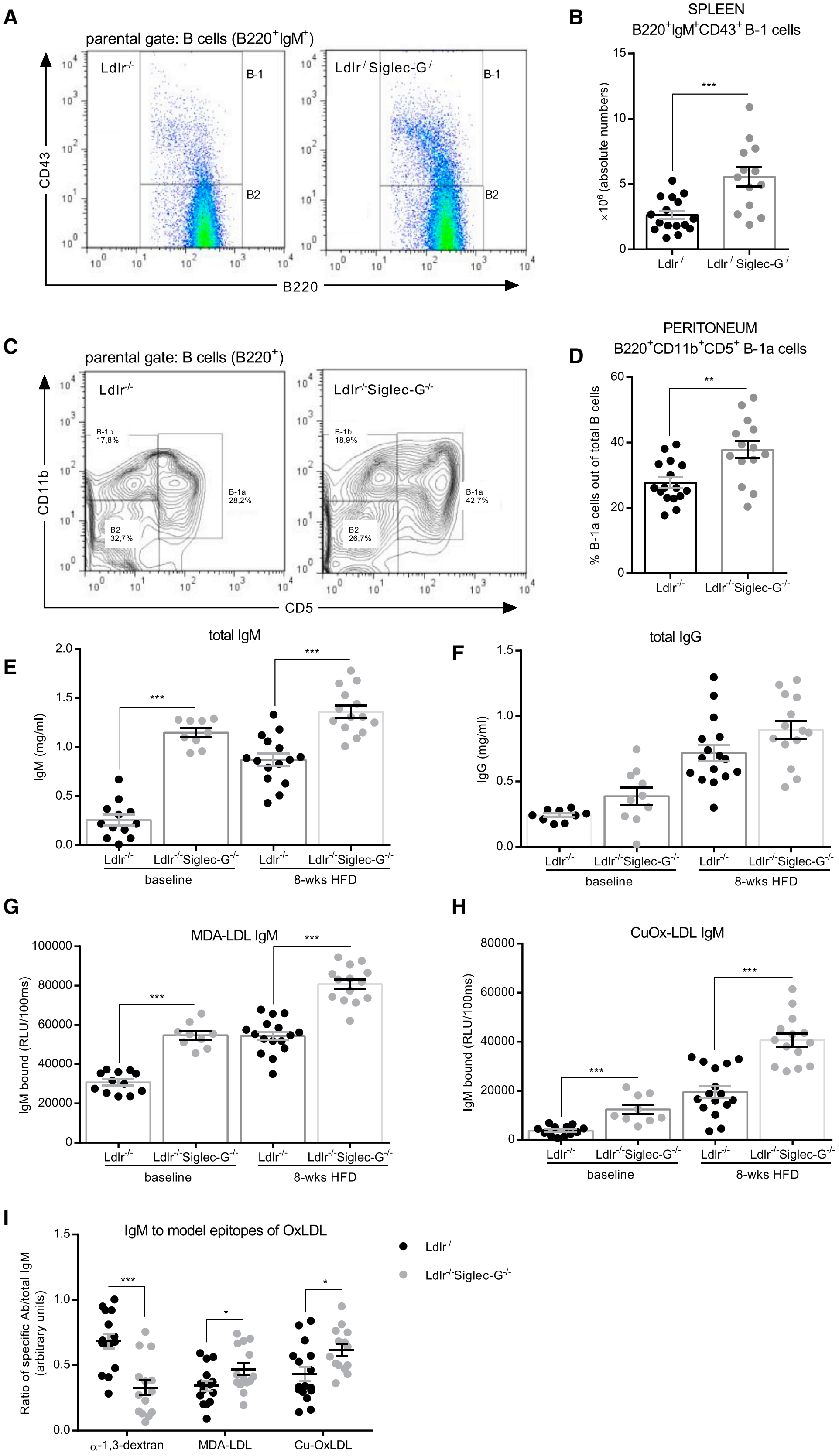

(legend on next page)

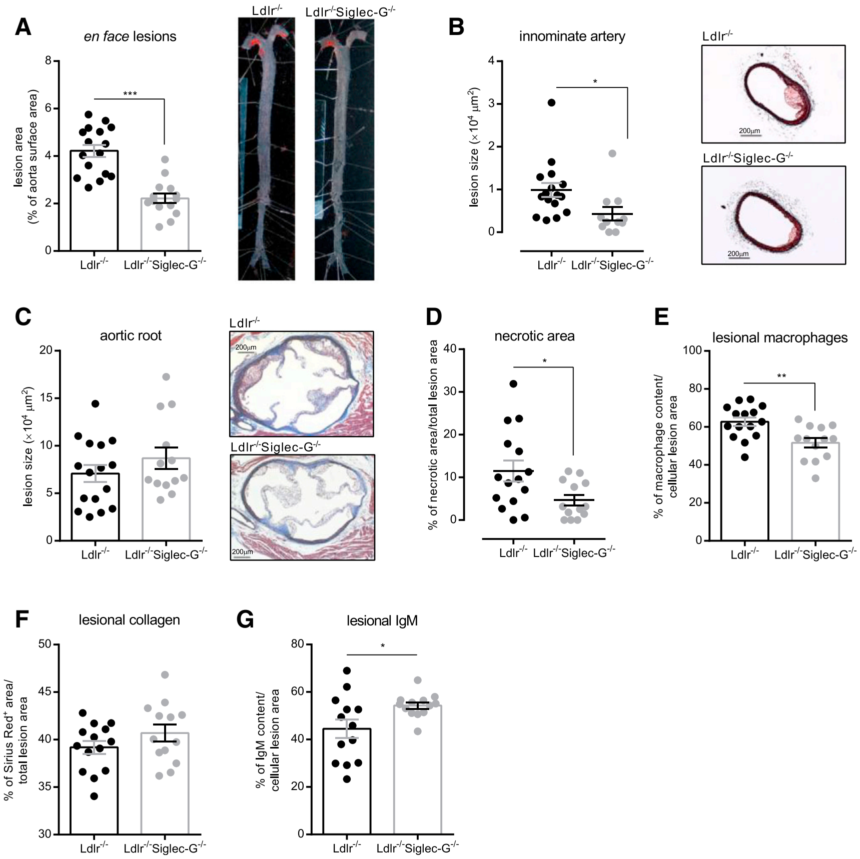

Figure 2. Siglec-G Deficiency Decreases Atherosclerosis in Cholesterol-Fed *Ldlr*$^{-/-}$ *Siglec-G*$^{-/-}$ Mice

(A) Quantitative analysis of atherosclerosis in the aorta. The data are expressed as percentage of Sudan IV stained area of the entire aorta. The representative pictures are shown on the right.

(B) Quantitative analysis of atherosclerotic lesions in cross-sections of innominate arteries. The values represent the average μm^2 of four sections throughout the innominate artery (300 μm). The images show representative Masson's trichrome stains. The original magnification is 50×, and the scale bar represents 200 μm.

(C) Quantification of atherosclerotic lesion size in cross-sections at the aortic origin. The values represent the average μm^2 of nine sections throughout the entire aortic origin (400 μm). The images show representative Masson's trichrome stains. The original magnification is 50X, and the scale bar represents 200 μm.

(D) Quantification of necrotic areas in cross-sections at the aortic origin. The values represent the percentages of necrotic areas of one section at 200 μm depth.

(E–G) Quantification of macrophage, collagen, and IgM content in cross-sections at the aortic origin.

(E) Values represent the percentage of mac-3$^+$ area per cellular lesion area of one section per mouse at 200 μm depth.

(F) Values represent the percentage of Sirius Red$^+$ area per total lesion area of one section per mouse at 200 μm depth.

(G) Values represent the percentage of IgM$^+$ area per cellular lesion area of one section per mouse at 180 μm depth. Shown are the data of *Ldlr*$^{-/-}$ and *Ldlr*$^{-/-}$*Siglec-G*$^{-/-}$ mice after 8 weeks of atherogenic diet. The symbols represent individual mice. The horizontal bars represent the mean of each group and errors bars represent SEM (*p ≤ 0.05, **p ≤ 0.01, and ***p ≤ 0.001).

See also Table 1.

engraftment of the respective bone marrow was confirmed by PCR of DNA isolated from bone marrow of recipients and of sorted splenic B and non-B cells to demonstrate selective Siglec-G deficiency (Figures S3A and S3B). In addition, the lack of Siglec-G expression on splenic B cells was confirmed by flow cytometry (Figure S3C).

Body weights, TC and TG levels in plasma, as well as frequencies of peripheral blood monocytes and B cells were not different between the experimental groups (Table 2; Figures S4A–S4C). Splenic B-1 cells as well as peritoneal B-1a cells were significantly increased in recipients of *Siglec-G*$^{-/-}$ bone marrow (whole and mixed bone marrow) compared to recipients of control bone marrow (Figures 3A and S4D; Table 2). Moreover, total plasma IgM levels were significantly increased in mice reconstituted with *Siglec-G*$^{-/-}$ and μMT+*Siglec-G*$^{-/-}$ bone marrow compared to their controls (Figure 3B), while total plasma IgG levels were not different (Figure S4E). IgM—but not IgG—levels to MDA-LDL and CuOx-LDL were

Figure 1. Siglec-G Deficiency in Cholesterol-Fed *Ldlr*$^{-/-}$ Mice Increases B-1a Cell-Derived IgM Antibodies

(A) Representative flow cytometry plots for splenic B220$^+$CD43$^-$ B2 cells and B220$^+$CD43$^+$ B-1 cell subpopulations. The parental gate was set on B220$^+$IgM$^+$ B cells.

(B) Absolute numbers of splenic B220$^+$IgM$^+$CD43$^+$ B-1 cells.

(C) Representative flow cytometry plots for B cell subpopulations of the peritoneal cavity. The parental gate was set on B220$^+$ B cells.

(D) Relative numbers of peritoneal B220$^+$CD5$^+$CD11b$^+$ B-1a cells out of total B cells.

(E and F) Quantification of total IgM (E) and total IgG (F) antibodies in plasma. The samples were diluted between 1:30,000 and 1:70,000 and measured in triplicates.

(G and H) Titers of MDA-LDL IgM (G) and CuOx-LDL IgM (H) in plasma were determined by ELISA at baseline and after 8 weeks of atherogenic diet. The samples were diluted between 1:100 and 1:500 and antibody binding was measured in triplicates. The data are expressed as relative light units (RLU) per 100 ms.

(I) RLU of IgM antibodies to MDA-LDL and CuOx-LDL as well as α-1,3-dextran (1:100) were normalized to total IgM concentrations and expressed as ratio of specific IgM per total IgM (a.u.). Shown are data of *Ldlr*$^{-/-}$ and *Ldlr*$^{-/-}$*Siglec-G*$^{-/-}$ mice after 8 weeks of atherogenic diet. The symbols represent individual mice. The horizontal bars represent the mean of each group and errors bars represent SEM (*p ≤ 0.05, **p ≤ 0.01, and ***p ≤ 0.001).

See also Figures S1 and S2.

Table 2. Parameters Overview of Experimental Groups: Bone Marrow Transplantation Study

	C57BL/6	*Siglec-G*$^{-/-}$	*μMT*+C57BL/6	*μMT+Siglec-G*$^{-/-}$
Ldlr$^{-/-}$ ←	(n = 10)	(n = 11)	(n = 13)	(n = 13)
Metabolic Parameters[a]				
Weights (g)	25.54 ± 0.63	25.15 ± 0.67	25.72 ± 0.49	25.29 ± 0.73
TC (mg/dL)	758.33 ± 79.47	821.59 ± 66.53	884.67 ± 92.20	925.96 ± 78.29
TG (mg/dL)	595.83 ± 49.43	567.05 ± 48.57	661.46 ± 73.94	585.58 ± 46.60
Atherosclerosis				
en face (% of aorta)	3.04 ± 0.38	0.59 ± 0.15***	3.11 ± 0.24	1.17 ± 0.34***
Aortic origin (× 10^4 μm^2/section)			4.84 ± 1.14	4.03 ± 1.13
Necrotic area (% of total lesion area)			5.20 ± 1.48	1.09 ± 0.66*
mac-3^+ (% of total area)			54.96 ± 6.57	38.60 ± 4.95*
Hepatic Inflammation				
Liver cholesterol (μg TC/μg protein)	0.22 ± 0.04	0.19 ± 0.03	0.23 ± 0.03	0.23 ± 0.03
Liver TG (μg TG/μg protein)	0.25 ± 0.04	0.21 ± 0.02	0.25 ± 0.03	0.22 ± 0.02
Liver free fatty acids (μg FFA/μg protein)	0.14 ± 0.01	0.12 ± 0.01	0.15 ± 0.01	0.15 ± 0.01
ALT (U/l)	109.1 ± 27.2	58.8 ± 11.5	47.6 ± 4.8	38.6 ± 3.0
Mac-1 (# positive cells/mm^2)	120.69 ± 13.08	86.72 ± 8.95*	100.68 ± 6.33	79.41 ± 5.50*
Ly6G (# positive cells/mm^2)	79.67 ± 5.82	58.58 ± 7.41*	74.40 ± 6.30	48.61 ± 4.04**
Serum Antibody Titers—10 Weeks HFD				
Total IgM (mg/ml)	0.47 ± 0.10	0.92 ± 0.11**	0.55 ± 0.10	1.07 ± 0.10***
MDA-LDL IgM (RLU/100ms)	23,939 ± 2,848	32,989 ± 1,848**	23,514 ± 1,706	30,045 ± 943**
CuOx-LDL IgM (RLU/100ms)	4,877 ± 951	12,566 ± 1,583***	5,714 ± 829	11,704 ± 1,045***
Total IgG (mg/ml)	0.47 ± 0.10	0.89 ± 0.10*	0.61 ± 0.09	0.98 ± 0.27
MDA-LDL IgG (RLU/100ms)	3,220 ± 633	6,126 ± 1,248	4,405 ± 701	4,012 ± 752
CuOx-LDL IgG (RLU/100ms)	2,885 ± 754	3,476 ± 769	1,827 ± 274	1,990 ± 452
Spleen (Absolute Numbers × 10^6)				
T cells ($CD3^+$)	41.93 ± 4.76	37.51 ± 3.86	67.76 ± 17.05	42.90 ± 8.13
B cells ($B220^+IgM^+$)	66.80 ± 9.44	74.18 ± 9.22	43.85 ± 12.07	47.46 ± 13.18
B1 cells ($B220^+IgM^+CD43^+$)	3.21 ± 0.38	6.15 ± 0.75**	2.82 ± 0.60	6.04 ± 1.32**
MZ B cells ($B220^+CD43^-CD23^-CD21^{hi}$)	1.98 ± 0.35	1.56 ± 0.26	4.58 ± 1.41	2.94 ± 1.28
T2/FO B cells ($B220^+CD43^-CD23^+CD21^+$)	46.04 ± 5.43	54.25 ± 7.49	23.47 ± 7.63	25.28 ± 8.42
T1 B cells ($B220^+CD43^-CD23^-CD21^{lo}$)	3.42 ± 0.77	3.46 ± 0.35	3.68 ± 2.40	2.24 ± 0.72
$B220^+CD43^-$ $CD23^-$ $CD21^+$	5.99 ± 1.18	5.48 ± 0.67	4.16 ± 1.05	5.38 ± 2.08
Peritoneal Cavity (% Viable Cells)				
B-1a cells ($B220^+CD11b^+CD5^+$) out of B cells	17.13 ± 1.40	31.85 ± 1.14***	25.18 ± 0.62	46.20 ± 1.07***
B-1b cells ($B220^+CD11b^+CD5^-$) out of B cells	14.17 ± 1.24	12.47 ± 0.97	19.45 ± 1.29	15.96 ± 0.83*
B2 cells ($B220^+CD11b^-CD5^-$) out of B cells	64.96 ± 2.32	48.47 ± 2.00***	47.40 ± 1.94	29.88 ± 1.06***
T cells ($B220^-CD5^+$) out of total	11.99 ± 1.19	15.07 ± 2.68	18.18 ± 5.04	13.97 ± 2.89

[a]Data are shown as mean ± SEM (* $p \leq 0.05$, ** $p \leq 0.01$, and *** $p \leq 0.001$).

also robustly and preferentially increased in recipient mice of *Siglec-G*$^{-/-}$ bone marrow (Figures 3C, 3D, and S4F–S4H). Importantly, total and B cell-selective deficiency of Siglec-G in the hematopoetic compartment resulted in a similar reduction of atherosclerotic lesion formation (Figure 3E) and a decreased lesional complexity with smaller necrotic areas and fewer lesional macrophages in the aortic root (Figures 3F–3H). This indicates that Siglec-G deficiency on B cells is primarily responsible for the protective effects in atherogenesis.

Siglec-G Deficiency in Mice Protects from Hepatic and Systemic Inflammation

We have recently demonstrated that *Ldlr*$^{-/-}$ mice develop hepatic inflammation when fed an atherogenic diet (Bieghs et al., 2012b). Therefore, we investigated the effect of Siglec-G deficiency on hepatic inflammation in these mice. Atherogenic diet feeding resulted in a robust steatosis of the liver of all experimental mice. We found no significant differences in hepatic cholesterol, hepatic TG, and hepatic free fatty acids between the experimental groups. Consistent with only minimal liver

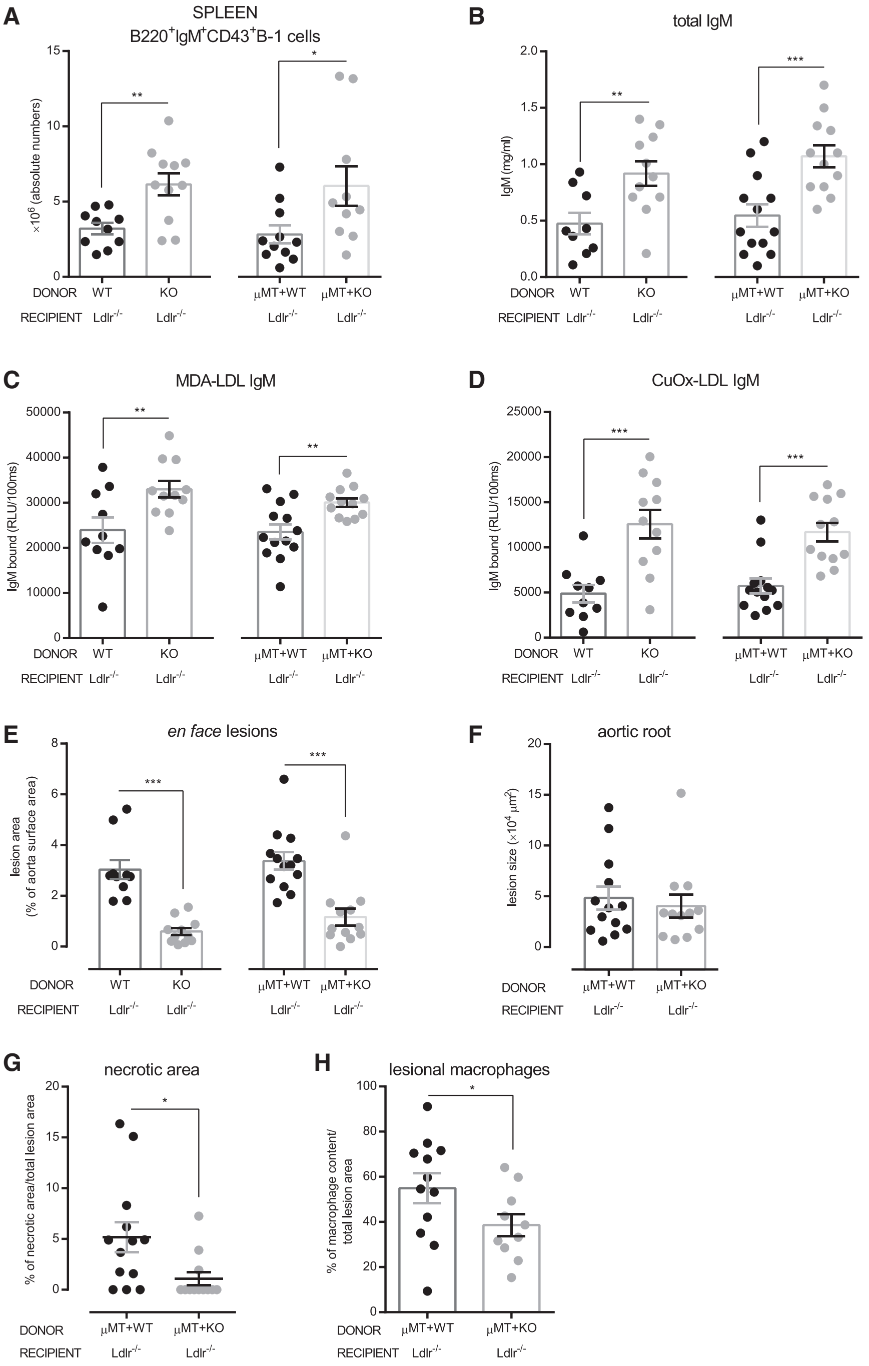

Figure 3. Selective Siglec-G Deficiency in B Cells Reduces the Development of Atherosclerosis

(A) Absolute numbers of splenic IgM$^+$B220$^+$CD43$^+$ B-1 cells.

(B) Quantification of total IgM in plasma. The samples were diluted 1:60,000 and measured in triplicates.

(C and D) Binding of IgM antibodies to (C) MDA-LDL and (D) CuOx-LDL was determined by ELISA. The plasma samples were diluted between 1:100 and 1:500 and antibody binding was measured in triplicates. The data are expressed as RLU/100 ms.

(E) Quantitative analysis of atherosclerosis (*en face*) in the entire aorta. The data are expressed as percentage of Sudan IV stained area of the entire aorta.

(F) Quantification of atherosclerotic lesion size in cross-sections at the aortic origin. The values represent the average μm^2 of nine sections throughout the entire aortic origin (400 µm).

(G) Quantification of necrotic areas in cross-sections at the aortic origin. The values represent percentages of necrotic areas of one section at 200 µm.

(H) Quantification of the macrophage content in cross-sections at the aortic origin. The values represent percentages of mac-3$^+$ area per total lesion area of one section per mouse at 200 µm depth. Shown are data of *Ldlr*$^{-/-}$ mice reconstituted with *Siglec-G*$^{+/+}$ [WT] versus *Siglec-G*$^{-/-}$ [KO] and µMT+*Siglec-G*$^{+/+}$ [WT] versus µMT+*Siglec-G*$^{-/-}$ [KO] bone marrow after 10 weeks of atherogenic diet. The symbols represent individual mice. The horizontal bars represent the mean of each group and errors bars represent SEM (*p $\leq$ 0.05, **p $\leq$ 0.01, and ***p $\leq$ 0.00).

See also Figures S3 and S4; Table 2.

damage in this model, alanine aminotransferase (ALT) levels were only moderately elevated, but also not different between the two groups (Tables 1 and 2). In contrast, immunohistochemical analyses of liver sections revealed a significantly reduced infiltration of Ly6G$^+$ neutrophils and mac-1$^+$ macrophages in the livers of *Ldlr*$^{-/-}$*Siglec-G*$^{-/-}$ mice compared to *Ldlr*$^{-/-}$ mice (Figures 4A and 4B). A similar significant reduction in neutrophil and macrophage infiltration was found in the livers of *Ldlr*$^{-/-}$ mice that were reconstituted with either *Siglec-G*$^{-/-}$ or µMT+*Siglec-G*$^{-/-}$ bone marrow (Figures S5A and S5B). To further define the effects on hepatic inflammation between *Ldlr*$^{-/-}$ and *Ldlr*$^{-/-}$*Siglec-G*$^{-/-}$ mice, we analyzed the expression of adhesion molecules, pro-inflammatory cytokines, and chemokines in the liver of these mice. In line with the reduced cell infiltration, expression of intercellular adhesion molecule (*Icam*) and vascular cell adhesion protein (*Vcam*), tumor necrosis factor-alpha (*Tnf-α*) and interleukin-18 (*Il-18*), C-C motif ligand 5 (*Ccl5*), C-X-C motif ligand 1 (*Cxcl1*), and C-X-C motif ligand 2 (*Cxcl2*) were significantly reduced in the livers of *Ldlr*$^{-/-}$*Siglec-G*$^{-/-}$ mice compared to *Ldlr*$^{-/-}$ mice (Figure 4C). A similar though less pronounced decrease of inflammatory gene expression was also observed in the livers of mice lacking Siglec-G in B cells only (Figure S5C). Thus, Siglec-G deficiency and Siglec-G deficiency on B cells reduces hepatic inflammation, suggesting an important role in the development of steatohepatitis.

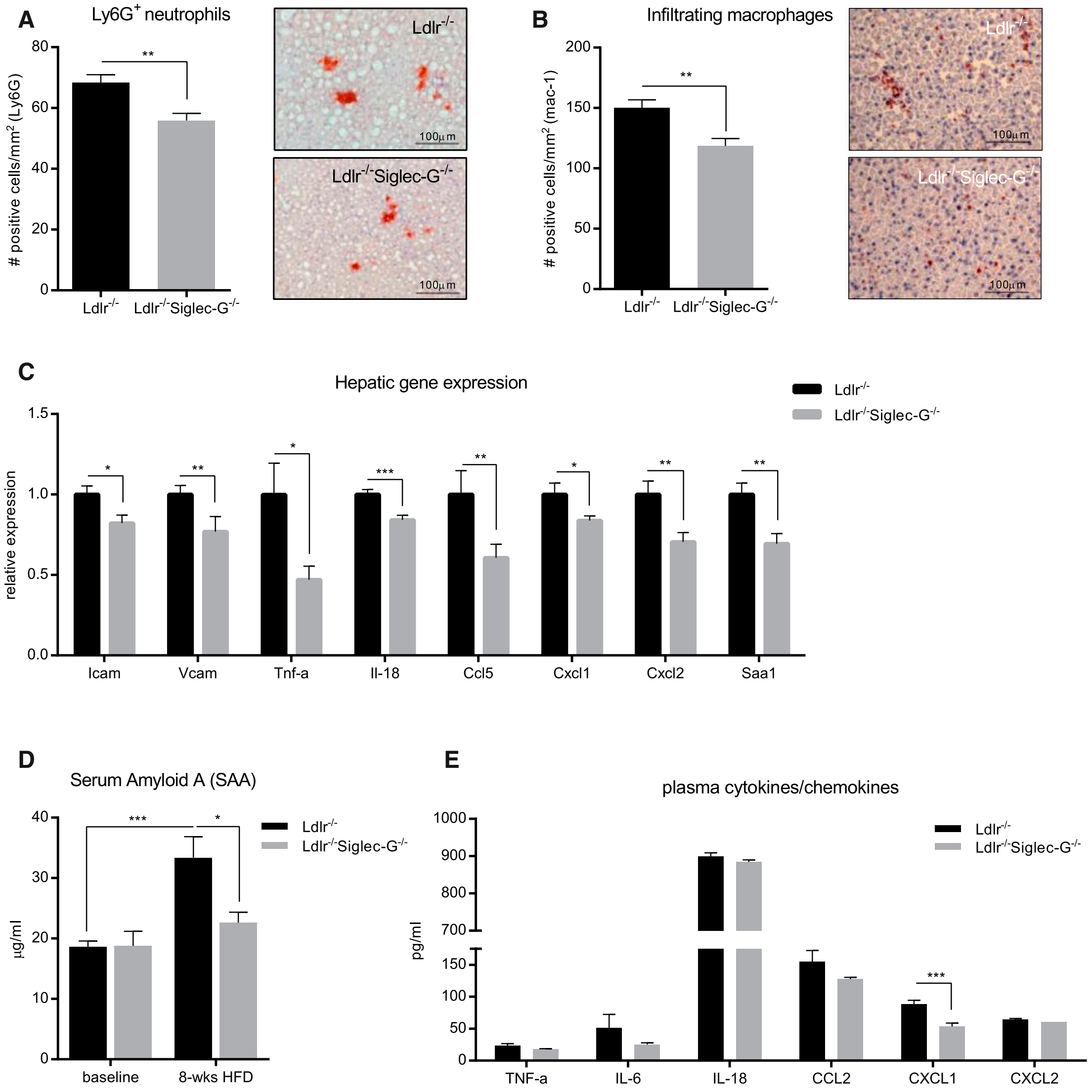

Figure 4. Siglec-G Deficiency Protects from Atherogenic Diet-Induced Hepatic and Systemic Inflammation

(A and B) Quantification of infiltrating neutrophils (A) and macrophages (B) in liver sections of *Ldlr*$^{-/-}$ versus *Ldlr*$^{-/-}$*Siglec-G*$^{-/-}$ mice after 8 weeks of atherogenic diet. The sections were stained with anti-Ly6G and anti-mac-1 antibody. The positively stained cells were counted and expressed as number of positive cells per mm^2. The representative pictures (original magnification 200× and the scale bar represents 100 μm) are shown on the right.

(C) Relative gene expression of *Icam, Vcam, Tnf-α, Il-18, Ccl5, Cxcl1, Cxcl2*, and *Saa1* mRNA in livers of *Ldlr*$^{-/-}$ versus *Ldlr*$^{-/-}$*Siglec-G*$^{-/-}$ mice after 8 weeks of atherogenic diet. The expression data of individual genes was normalized to the house-keeping gene S12 and expressed relative to the expression in *Ldlr*$^{-/-}$ control mice.

(D) Quantification of SAA levels in plasma of *Ldlr*$^{-/-}$ versus *Ldlr*$^{-/-}$*Siglec-G*$^{-/-}$ mice at baseline and after 8 weeks of atherogenic diet. The samples were diluted 1:300 and measured in triplicates.

(E) Quantification of TNFα, IL-6, IL-18, CCL2, CXCL1, and CXCL2 in plasma of *Ldlr*$^{-/-}$ versus *Ldlr*$^{-/-}$*Siglec-G*$^{-/-}$ mice after 8 weeks of atherogenic diet by multiplex assay. The samples were diluted 1:2. The data represent mean ± SEM of 9–13 mice per group (*p ≤ 0.05, **p ≤ 0.01, and ***p ≤ 0.001).

See also Figure S5.

To assess systemic markers of inflammation in these mice, we quantified hepatic mRNA expression and the circulating levels of serum amyloid A (SAA), which in mice represents an acute phase protein that is induced during chronic inflammation (Uhlar and Whitehead, 1999). Expression of *Saa1* was significantly reduced in the livers of *Ldlr*$^{-/-}$*Siglec-G*$^{-/-}$ mice compared to *Ldlr*$^{-/-}$ mice (Figure 4C). Moreover, while plasma SAA levels increased after 8 weeks of atherogenic diet in *Ldlr*$^{-/-}$, this was not the case for *Ldlr*$^{-/-}$*Siglec-G*$^{-/-}$ mice (Figure 4D). SAA levels in *Ldlr*$^{-/-}$ mice that were reconstituted with μMT+*Siglec-G*$^{-/-}$ or μMT+*wild-type* (*WT)* bone marrow after 10 weeks of atherogenic diet were lower in both groups, consistent with a more moderate degree of inflammation, but still showed a tendency toward reduced levels as a result of

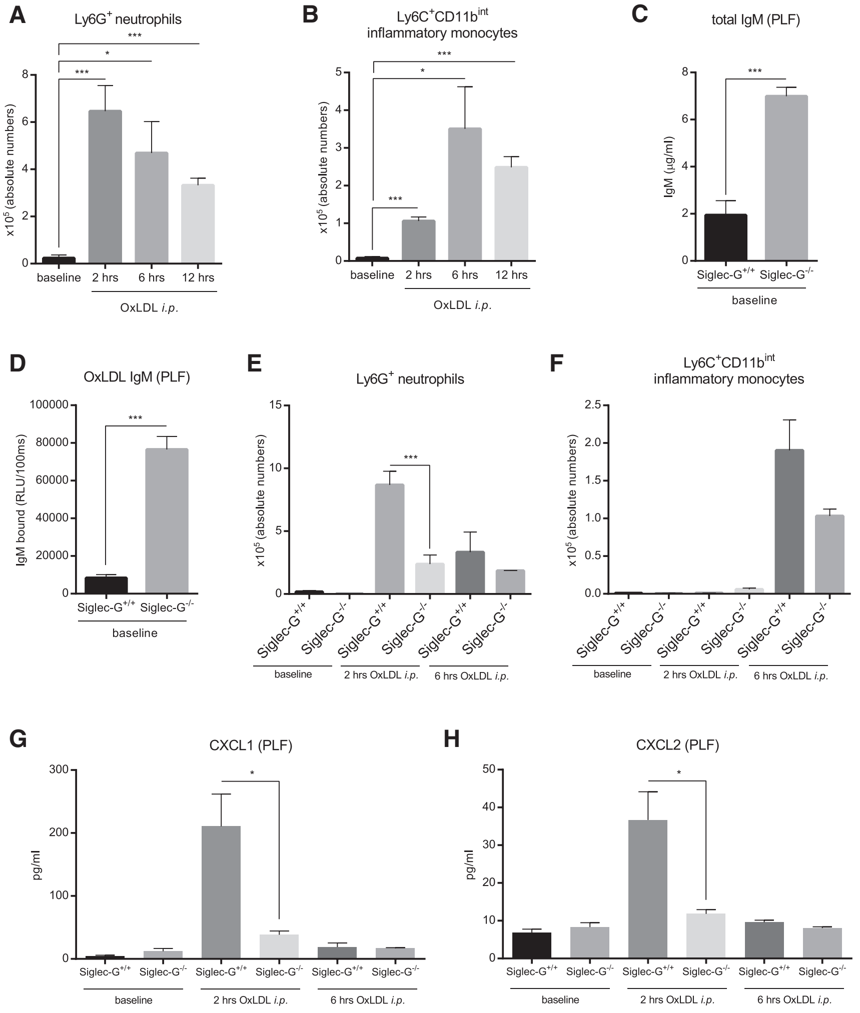

Figure 5. OxLDL-Induced Sterile Peritonitis Is Suppressed by Siglec-G Deficiency

(A and B) Quantification of absolute numbers of Ly6G$^+$ neutrophils (A) and Ly6C^{hi} inflammatory monocytes (B) in the PLF of C57BL/6 mice at baseline, as well as 2, 6, and 12 hr after OxLDL injections.

(C) Quantification of total IgM in the PLF of *Siglec-G*$^{+/+}$ and *Siglec-G*$^{-/-}$ mice. The samples were diluted 1:20 and measured in triplicates.

(D) Binding of IgM to CuOx-LDL in the PLF of *Siglec-G*$^{+/+}$ and *Siglec-G*$^{-/-}$ mice was determined by ELISA. The samples were undiluted and antibody binding was measured in triplicates. The data are expressed as relative RLU/100 ms.

(E and F) Quantification of absolute numbers of Ly6G$^+$ neutrophils (E) and Ly6C^{hi} monocytes (F) in the PLF of *Siglec-G*$^{+/+}$ and *Siglec-G*$^{-/-}$ mice at baseline, 2, and 6 hr after i.p. injection of OxLDL.

(G and H) Quantification of CXCL1 (G) and CXCL2 (H) in the PLF of *Siglec-G*$^{+/+}$ and *Siglec-G*$^{-/-}$ mice at indicated time points by ELISA. The samples were undiluted and measured in duplicates. The data represent mean ± SEM of 3–7 mice per group and are a representative example of three individual experiments (*$p \leq 0.05$, **$p \leq 0.01$, and ***$p \leq 0.001$). See also Figures S6 and S7.

Siglec-G deficiency (Figure S5D). To better characterize the inflammatory responses in these mice, we quantified the plasma levels of cytokines (TNF-α, IL-6, and IL-18) and chemokines (CCL2, CXCL1, and CXCL2) that have been previously identified to contribute to different pathogenic responses in atherosclerosis and hepatic inflammation (Ait-Oufella et al., 2011; Weber and Noels, 2011). Of all cytokines and chemokines tested, plasma levels of CXCL1 were significantly reduced both in *Ldlr*$^{-/-}$*Siglec-G*$^{-/-}$ mice (Figure 4E) and *Ldlr*$^{-/-}$ mice lacking Siglec-G in B cells only compared to their respective controls (Figure S5E). Notably, Cxcl1 has been shown to be prominently induced in macrophages and endothelial cells stimulated with OxLDL and mediates the recruitment of neutrophils and monocytes (Stewart et al., 2010; Berliner et al., 1990).

Thus, atherogenic diet is associated with increased inflammation in *Ldlr*$^{-/-}$ mice, and this effect is suppressed in mice lacking Siglec-G. Collectively, these data point to a strong anti-inflammatory effect of Siglec-G deficiency in diet-induced hepatic and systemic inflammation.

Siglec-G Deficiency Protects from OxLDL-Induced Sterile Peritonitis

Because we have previously shown that OSE-specific NAb inhibit pro-inflammatory effects of epitopes of OxLDL (Tsiantoulas et al., 2015; Imai et al., 2008), which is considered a major driver of vascular and hepatic inflammation (Miller et al., 2011; Walenbergh et al., 2013), we evaluated the effect of Siglec-G deficiency in an OxLDL-induced sterile inflammation model in vivo. For this, we first established a sterile peritonitis model that allowed us to monitor the recruitment of neutrophils and monocytes in the peritoneal lavage fluid (PLF) of WT C57BL/6 mice 2, 6, and 12 hr after they had received an intraperitoneal (i.p.) injection of OxLDL. Compared to baseline, mice injected with OxLDL exhibited a marked recruitment of Ly-6G$^+$ neutrophils into the peritoneal cavity already after 2 hr, which remained significantly different, but gradually declined after 6 and 12 hr (Figures 5A, S6, and S7A). Similarly, peritoneal recruitment of Ly6C$^+$CD11b^{int} inflammatory monocytes was also induced following i.p. injection of OxLDL, but only peaked 6 hr after the injection, consistent with the delayed recruitment of monocytes during inflammatory processes (Figures 5B and S7A).

To investigate the anti-inflammatory effect of natural IgM associated with Siglec-G deficiency, we compared the dynamics of inflammatory cell recruitment during OxLDL-induced peritonitis in *Siglec-G*$^{+/+}$ and *Siglec-G*$^{-/-}$ mice, respectively. Consistent with the dramatic increase of IgM antibodies in the plasma of *Siglec-G*$^{-/-}$ mice, we observed >3-fold higher levels of total and >9-fold higher levels of OxLDL-specific IgM in

the PLF of *Siglec-G*$^{-/-}$ mice at baseline (Figures 5C and 5D). At baseline, the numbers of peritoneal macrophages, which represent the cellular population that is primarily involved in responding to OxLDL, were not significantly different between *Siglec-G*$^{+/+}$ and *Siglec-G*$^{-/-}$ mice (Figure S7C). *Siglec-G*$^{-/-}$ mice showed a significantly reduced recruitment of neutrophils 2 hr after i.p. injection of OxLDL compared to *Siglec-G*$^{+/+}$ mice, while after 6 hr, no differences between the two groups were observed (Figure 5E). Consistent with this, OxLDL-induced secretion of CXCL1 and CXCL2 was nearly absent in the peritoneal cavity of *Siglec-G*$^{-/-}$ mice at this time point (Figures 5G and 5H). At 6 hours after i.p. injection of OxLDL, the recruitment of Ly-6C^{+}CD11b^{int} inflammatory monocytes appeared lower in *Siglec-G*$^{-/-}$ mice (Figure 5F). In contrast, Siglec-G deficiency had no effect on neutrophil recruitment in response to injection of thioglycollate (another sterile trigger), indicating that Siglec-G-deficient mice do not have defective inflammatory responses in general and demonstrating specificity for inflammatory responses triggered by OSE (Figure S7D). In summary, Siglec-G deficiency results in a significant inhibition of the inflammatory response to OxLDL in vivo. The markedly reduced infiltration of neutrophils in *Siglec-G*$^{-/-}$ mice in response to i.p. injection of OxLDL indicates direct neutralization of its pro-inflammatory moieties and more efficient clearance mediated by OSE-specific IgM.

DISCUSSION

In this study, we demonstrate a pathogenic role for Siglec-G in atherosclerosis and hepatic inflammation. We could show that Siglec-G deficiency in atherosclerosis-prone *Ldlr*$^{-/-}$ mice leads to a marked reduction of atherosclerotic lesion burden as well as decreased hepatic inflammation. This effect is specific for Siglec-G deficiency in B cells that results in higher B-1 cell numbers and a robust and preferential increase in OSE-specific IgM antibodies, which neutralize OxLDL-induced inflammation in vivo.

Siglec-G is primarily expressed on B cells, with slightly higher expression levels on B-1a cells than on conventional B2 cells (Hoffmann et al., 2007), but it has also been shown to be expressed on dendritic cells and eosinophils (Hutzler et al., 2014; Pfrengle et al., 2013). A suppressive role of Siglec-G in non-B cells has been suggested in drug-induced liver damage, as Siglec-G deficiency results in increased production of pro-inflammatory cytokines, including IL-6, TNF-α, and MCP-1, following stimulation with DAMPs that are released in this setting (Chen et al., 2009). Nevertheless, our data clearly identify an anti-inflammatory effect of Siglec-G deficiency in cholesterol-fed *Ldlr*$^{-/-}$ mice as documented by reduced hepatic expression of adhesion molecules, pro-inflammatory cytokines, and chemokines as well as lower CXCL1 levels in plasma. Moreover, our data demonstrate Siglec-G deficiency to be protective in a model of OxLDL-induced peritonitis, while it has been shown to aggravate inflammation in cecal ligation and puncture-induced polybacterial peritonitis. These detrimental effects of Siglec-G deficiency have been shown to be dependent on its interaction with the glycosylphosphatidylinositol (GPI)-anchored co-stimulatory protein CD24 (Chen et al., 2011). CD24 has been shown to recognize DAMPs and to allow Siglec-G to suppress pro-inflammatory signaling via pattern-recognition receptors, which can be disrupted by bacterial sialidases, but not by LPS. In our study, Siglec-G deficiency conferred a protective effect against another type of sterile trigger, OxLDL, as Siglec-G-deficient mice exhibited decreased inflammation in an OxLDL-induced peritonitis model. Presumably, the sterile inflammation induced by OxLDL also does not disrupt the interaction of CD24 and Siglec-G, suggesting that Siglec-G deficiency protects via other mechanisms than CD24.

It has also been shown that RNA viruses evade anti-viral defenses by upregulating Siglec-G in macrophages and dendritic cells, resulting in diminished production of type I interferons that are essential in the host defense against viruses (Chen et al., 2013). As a consequence of this, Siglec-G deficiency results in increased production of type I interferons, which notably have been shown to promote atherosclerosis in several studies (Ait-Oufella et al., 2011). Because we have shown that Siglec-G deficiency protects from atherosclerosis, this pathway seems to be not relevant in this setting. In line with this, stimulation of peritoneal and bone-marrow derived macrophages with OxLDL does not induce Siglec-G expression (data not shown). Taken together, our data argue against a role of Siglec-G in myeloid cells with respect to atherosclerosis and hepatic inflammation. Moreover, we provide clear evidence that the protective effect of Siglec-G deficiency is mediated by B cells, indicating that the function of Siglec-G on B cells is dominant. In B cells, Siglec-G is expressed in all stages throughout B cell development (Pfrengle et al., 2013; Hutzler et al., 2014). Therefore, its functional role in autoimmunity has been studied, particularly in aging mice (Müller et al., 2015). Old *Siglec-G*$^{-/-}$ mice have been shown to exhibit an increased expression of activation markers on T cells and conventional B2 cells, suggesting a more general over-activation of the adaptive immune system in these settings. However, the effects described in our study are more consistent with the increased production of protective B-1 cell-derived NAb by Siglec-G-deficient mice. In line with previous data, we demonstrate that Siglec-G deficiency also resulted in a dramatic expansion of peritoneal B-1a as well as splenic B-1 cells in atherosclerotic mice. FO B cells and MZB cells remained unchanged in *Ldlr*$^{-/-}$*Siglec-G*$^{-/-}$ mice as well as in the bone marrow chimeric mice. Thus, our data support a protective role of B-1 cells in atherosclerosis (Tsiantoulas et al., 2014). Notably, we did observe a robust increase of the recently described IRA B cells in the spleens of atherosclerotic *Ldlr*$^{-/-}$*Siglec-G*$^{-/-}$ mice. These cells have been suggested to originate from B-1a cells and have been shown to promote atherosclerotic lesion formation (Hilgendorf et al., 2014). Thus, although our data are in line with a B-1 cell origin of IRA B cells, the protective effects of B-1 cells dominate in atherosclerotic mice lacking Siglec-G. Previous studies demonstrated an atheroprotective effect of adoptive transfer of B-1a cells into splenectomized *Apoe*$^{-/-}$ mice and of B-1b cells into *Rag-1*$^{-/-}$*Apoe*$^{-/-}$ mice (Kyaw et al., 2011; Rosenfeld et al., 2015). These studies address the role of B-1 cells only in immune-compromised mice on top of existing differences in various immune cell populations, such as lack of splenocytes or total lymphocytes as a result of splenectomy or Rag-1 deficiency, respectively. However, they do not provide a conclusive answer on

the role of B-1 cells in atherosclerosis in an intact host. Our data provide clear and direct genetic evidence that expansion of B-1a cells in intact mice mediates atheroprotection.

One of the major functions of B-1 cells is the secretion of natural IgM antibodies, which have been suggested to mediate the atheroprotective effect of B-1 cells. Kyaw et al. (2011) showed that accelerated lesion formation in splenectomized *Ldlr*$^{-/-}$ mice, which exhibit diminished peritoneal B-1a cell numbers and reduced serum IgM, is reversed by the adoptive transfer of B-1a cells. This effect was dependent on the capacity of B-1a cells to secrete IgM, as adoptive transfer of B-1 cells from secreted *IgM*$^{-/-}$ donors did not have any effect. The atheroprotective role of NAbs is further supported by the fact that *Ldlr*$^{-/-}$ mice deficient in secreted IgM develop increased atherosclerosis compared to *Ldlr*$^{-/-}$ control mice (Lewis et al., 2009). These data also suggested that increasing IgM titers above and beyond normal plasma levels could confer atheroprotection. Interestingly, hypercholesterolemia raises plasma IgM levels, presumably as part of an endogenous defense mechanism against increased accumulation of metabolic waste products (Khoo et al., 2015). However, this protective response is not sufficient to prevent the formation of atherosclerotic plaques and hepatic inflammation. Deficiency of Siglec-G results in an even greater increase of IgM antibodies despite excessive hypercholesterolemia, and thus Siglec-G may be an attractive target for therapeutic intervention, for example, by blocking antibodies.

Natural IgM exhibit house-keeping functions by promoting the clearance of dying cells and cellular debris (Avrameas, 1991; Notkins, 2004). These properties of natural IgM are in part mediated by their ability to recognize OSE, which are also present in OxLDL (Miller et al., 2011). We have previously shown that immunization of high-fat diet-fed *Ldlr*$^{-/-}$ mice with heat-killed pneumococci led to the expansion of a natural IgM clone recognizing OxLDL (T15/E06) and significantly decreased atherosclerotic lesion formation and hepatic inflammation (Binder et al., 2003; Bieghs et al., 2012b). These data suggested that anti-OxLDL IgM protect from atherosclerosis and liver inflammation. Indeed, epidemiological studies in humans indicate that high levels of OxLDL-specific IgM are associated with a lower incidence of cardiovascular disease (Tsiantoulas et al., 2014). Of note, Siglec-G deficiency, which results in atheroprotection, leads to a preferential increase of IgM with specificity for different epitopes of OxLDL in plasma.

Several mechanisms exist by which OxLDL-specific IgM exhibit atheroprotective properties, including their ability to inhibit foam cell formation by blocking scavenger receptor-mediated uptake of OxLDL by macrophages (Tsiantoulas et al., 2014). In addition, anti-OxLDL IgM prevent the accumulation of apoptotic cells by promoting their uptake by macrophages (Ogden et al., 2005; Chou et al., 2009). In line with this, we show smaller necrotic areas in lesion of *Ldlr*$^{-/-}$*Siglec-G*$^{-/-}$ mice. Moreover, anti-OxLDL IgM inhibit inflammation by components of OxLDL in vitro (Imai et al., 2008). In agreement with a neutralizing effect of anti-OxLDL IgM, we now show reduced CXCL1 and CXCL2 secretion as well as neutrophil infiltration into the peritoneum in vivo following injection of OxLDL into *Siglec-G*$^{-/-}$ mice, which have >9-fold higher levels of anti-OxLDL IgM in the peritoneal cavity compared to WT controls. Moreover, plasma levels of CXCL1 were significantly reduced in Siglec-G-deficient atherosclerotic mice. OxLDL has been shown to induce Cxcl1 expression in macrophages via a cooperation of CD36/TLR4/TLR6, and CXCL1 represents a key mediator of leukocyte recruitment in atherosclerosis (Stewart et al., 2010). Moreover, both macrophage CD36 and TLR-4 expression have been shown to promote atherogenesis and hepatic inflammation. Thus, increased natural IgM with specificity for OxLDL have the capacity to inhibit atherosclerotic lesion formation and hepatic inflammation in Siglec-G-deficient mice by neutralizing the pro-inflammatory properties of OxLDL.

In conclusion, we provide evidence that expansion of natural IgM antibodies beyond physiological plasma levels protect from atherosclerosis and hepatic inflammation. Exploiting these mechanisms, for example, by blocking the inhibitory effect of Siglec-G, may represent a therapeutic approach to enhance and strengthen the endogenous defense mechanisms to protect from cardiovascular disease and associated metabolic disorders, such as non-alcoholic steatohepatitis.

EXPERIMENTAL PROCEDURES

An expanded version of experimental procedures can be found in Supplemental Information.

Animals and Intervention Studies

All experimental protocols were approved by the institutional animal experimentation committee and the Austrian Ministry of Science. All mice were on a C57BL/6 background. *Ldlr*$^{-/-}$ and *μMT* mice were purchased originally from The Jackson Laboratories (Bar Harbor, Maine, USA). The generation of C57BL/6 *Siglec-G*$^{-/-}$ mice has been described elsewhere (Müller et al., 2015). *Siglec-G*$^{-/-}$ mice were further crossed with *Ldlr*$^{-/-}$ mice to obtain *Ldlr*$^{-/-}$*Siglec-G*$^{-/-}$ and *Ldlr*$^{-/-}$*Siglec-G*$^{+/+}$ mice. For intervention studies, three cohorts of 12-week-old male *Ldlr*$^{-/-}$*Siglec-G*$^{-/-}$ mice (n = 14) and *Ldlr*$^{-/-}$*Siglec-G*$^{+/+}$ (n = 16) littermate controls were fed an atherogenic diet containing 21% milk fat and 0.2% cholesterol (TD88137, Ssniff Spezialdiäten) for 8 weeks. Bone marrow transplantation studies were performed as previously described (Binder et al., 2004; Sage et al., 2012; Fillatreau et al., 2002) and as described in Supplemental Information.

Analysis of Atherosclerotic Lesions

The extent of atherosclerosis was assessed in a blinded fashion in *en face* preparations of the entire aorta and in cross-sections through the aortic origin by computer-assisted image analysis as previously described (Binder et al., 2003, 2004; Cardilo-Reis et al., 2012).

Liver Histology

The left lobes of livers were harvested and four equal pieces were snap frozen in liquid nitrogen for further analyses. Frozen liver sections (7 μm) were stained for the presence of infiltrating macrophages and neutrophils as described previously (Bieghs et al., 2012a). For the presence of neutrophils, sections were stained with mouse anti-Ly6G (1:50; BD PharMingen). Cell nuclei were counterstained with hematoxylin (Klinipath). Pictures were taken with a Nikon digital camera DMX1200 and ACT-1 v2.63 software (Nikon). The number of positive-stained cells was counted in six pictures (200 ×) per liver per mouse to determine the level of liver inflammation.

Flow Cytometry

Peritoneal exudate cells (PEC), splenocytes, bone marrow cells, and blood leukocytes were harvested as previously described (Cardilo-Reis et al., 2012) and stained as indicated in Supplemental Information. Stained cell populations were analyzed by multiparameter flow cytometry using a BD FACSCalibur (BD Bioscience) or BD FACS Fortessa, respectively. Dead cells and doublets were excluded by forward- and side-scatter and data were

analyzed using the FlowJo v10 data analysis software (Tree Star). Criteria used to identify the various cell populations are provided in Table 1.

Plasma Antibody and Protein Analyses

Total IgM levels were measured by a chemiluminescent-based sandwich ELISA as described previously (Chou et al., 2009). Total IgG levels were measured in plasma using a commercially available ELISA (Mouse IgG total ELISA Ready-SET-Go!, eBioscience). Cu^{2+}-OxLDL (CuOx-LDL) and MDA-LDL were prepared as described previously (Chou et al., 2009). α-1,3-dextran was a gift of John F. Kearney (University of Alabama at Birmingham, Alabama, USA). Antigen-specific antibody titers were measured by chemiluminescent ELISA as previously described (Binder et al., 2003). OxLDL-specific IgM per total IgM ratios were calculated based on the measurements of specific IgM at non-saturating dilutions (expressed as RLU/100 ms) and total IgM quantities of each individual sample. Data are expressed as a.u. of these ratios. SAA levels were measured in plasma diluted 1:300 using a commercially available ELISA for mouse SAA (Tridelta Development).

Measurement of Chemokines/Cytokines in Plasma and PLF

A panel of chemokines and cytokines (IL-6, TNF-α, CCXL1, CXCL2, CCL2, and IL-18) was measured in mouse plasma (1:2 dilution) with ProcartaPlex Multiplex Immunoassays (eBioscience) according to the manufacturer's protocol. Analysis was performed with the xMAP Technology by Luminex. Levels of CXCL1 and CXCL2 in the PLF were determined by using mouse CXCL1/KC DuoSet ELISA and Mouse CXCL2/MIP-2 DuoSet ELISA, respectively according to the manufacturer's protocol (R&D Systems).

Murine Sterile Peritonitis

Siglec-G$^{-/-}$ mice and WT littermate controls (8–12 weeks of age) were injected i.p. with sterile CuOx-LDL (tested for endotoxin levels by chromogenic Limulus amoebocyte assay; <0.05 ng LPS/mg protein) suspended in 600 μl of sterile PBS-BSA (25 μg/gram body weight) or a comparable volume of sterile thioglycollate (Lipid MAPS) (25 μl/g body weight). At selected time points, mice were sacrificed, and PEC were collected by lavaging the peritoneum with 6 ml of sterile PBS+1% BSA. PEC were pelleted for 10 min at 300 g, and processed for flow cytometry, as described above. Cell-free lavage fluid was stored in aliquots and further processed for ELISA measurements.

Statistical Analysis

Data were analyzed using GraphPad Prism version 6.0 for Windows (GraphPad Software). Normal distribution of data was assessed, and statistical analyses were performed by unpaired Student's t-test to determine statistical significance between the groups. Data points, which were more than 2 × SD of the mean, were excluded as statistical outliers. Exclusion of these values did not change the significance of the results. Data are presented as the mean ± SEM and considered significant at $p \leq 0.05$ (*$p \leq 0.05$, **$p \leq 0.01$, and ***$p \leq 0.001$, respectively).

SUPPLEMENTAL INFORMATION

Supplemental Information includes Supplemental Experimental Procedures and seven figures and can be found with this article online at http://dx.doi.org/10.1016/j.celrep.2016.02.027.

AUTHOR CONTRIBUTIONS

S.G. designed and performed experiments, analyzed and interpreted data, and wrote the manuscript. T.H. and D.T. performed experiments, analyzed and interpreted data, and critically revised the manuscript. M.O.-K. and L.G. performed experiments. J.L.W. provided intellectual input and critically revised the manuscript. Z.M., R.S.-S., and L.N. provided technical and material support and critically revised the manuscript. C.J.B. conceived, designed, and supervised the study and drafted and critically revised the manuscript.

ACKNOWLEDGMENTS

The authors thank Robert Kralovics, Sylvia Knapp, Riem Gawish, and Anastasiya Hladik for technical and material support. We are grateful to Georg Obermayer for help with the illustrations. This work was supported by grants of the Austrian Academy of Sciences (C.J.B.), the Austrian Science Fund (SFB-54; C.J.B.), the European Union (FP7 programme VIA; C.J.B.), the German Research Foundation (TRR130, L.N.), the Fondation Leducq (C.J.B., Z.M., and J.L.W.), and The Netherlands Organisation for Scientific Research (NWO) (Vidi 016.126.327, R.S.-S.).

Received: August 17, 2015
Revised: December 21, 2015
Accepted: February 1, 2016
Published: March 3, 2016

REFERENCES

Ait-Oufella, H., Herbin, O., Bouaziz, J.D., Binder, C.J., Uyttenhove, C., Laurans, L., Taleb, S., Van Vré, E., Esposito, B., Vilar, J., et al. (2010). B cell depletion reduces the development of atherosclerosis in mice. J. Exp. Med. *207*, 1579–1587.

Ait-Oufella, H., Taleb, S., Mallat, Z., and Tedgui, A. (2011). Recent advances on the role of cytokines in atherosclerosis. Arterioscler. Thromb. Vasc. Biol. *31*, 969–979.

Avrameas, S. (1991). Natural autoantibodies: from "horror autotoxicus" to "gnothi seauton". Immunol. Today *12*, 154–159.

Baumgarth, N., Tung, J.W., and Herzenberg, L.A. (2005). Inherent specificities in natural antibodies: a key to immune defense against pathogen invasion. Springer Semin. Immunopathol. *26*, 347–362.

Berliner, J.A., Territo, M.C., Sevanian, A., Ramin, S., Kim, J.A., Bamshad, B., Esterson, M., and Fogelman, A.M. (1990). Minimally modified low density lipoprotein stimulates monocyte endothelial interactions. J. Clin. Invest. *85*, 1260–1266.

Bieghs, V., Rensen, P.C., Hofker, M.H., and Shiri-Sverdlov, R. (2012a). NASH and atherosclerosis are two aspects of a shared disease: central role for macrophages. Atherosclerosis *220*, 287–293.

Bieghs, V., van Gorp, P.J., Walenbergh, S.M., Gijbels, M.J., Verheyen, F., Buurman, W.A., Briles, D.E., Hofker, M.H., Binder, C.J., and Shiri-Sverdlov, R. (2012b). Specific immunization strategies against oxidized low-density lipoprotein: a novel way to reduce nonalcoholic steatohepatitis in mice. Hepatology *56*, 894–903.

Binder, C.J., Hörkkö, S., Dewan, A., Chang, M.K., Kieu, E.P., Goodyear, C.S., Shaw, P.X., Palinski, W., Witztum, J.L., and Silverman, G.J. (2003). Pneumococcal vaccination decreases atherosclerotic lesion formation: molecular mimicry between Streptococcus pneumoniae and oxidized LDL. Nat. Med. *9*, 736–743.

Binder, C.J., Hartvigsen, K., Chang, M.K., Miller, M., Broide, D., Palinski, W., Curtiss, L.K., Corr, M., and Witztum, J.L. (2004). IL-5 links adaptive and natural immunity specific for epitopes of oxidized LDL and protects from atherosclerosis. J. Clin. Invest. *114*, 427–437.

Bökers, S., Urbat, A., Daniel, C., Amann, K., Smith, K.G., Espéli, M., and Nitschke, L. (2014). Siglec-G deficiency leads to more severe collagen-induced arthritis and earlier onset of lupus-like symptoms in MRL/lpr mice. J. Immunol. *192*, 2994–3002.

Cardilo-Reis, L., Gruber, S., Schreier, S.M., Drechsler, M., Papac-Milicevic, N., Weber, C., Wagner, O., Stangl, H., Soehnlein, O., and Binder, C.J. (2012). Interleukin-13 protects from atherosclerosis and modulates plaque composition by skewing the macrophage phenotype. EMBO Mol. Med. *4*, 1072–1086.

Chan, V.W., Meng, F., Soriano, P., DeFranco, A.L., and Lowell, C.A. (1997). Characterization of the B lymphocyte populations in Lyn-deficient mice and the role of Lyn in signal initiation and down-regulation. Immunity *7*, 69–81.

Chang, M.K., Bergmark, C., Laurila, A., Hörkkö, S., Han, K.H., Friedman, P., Dennis, E.A., and Witztum, J.L. (1999). Monoclonal antibodies against oxidized low-density lipoprotein bind to apoptotic cells and inhibit their phagocytosis by elicited macrophages: evidence that oxidation-specific epitopes mediate macrophage recognition. Proc. Natl. Acad. Sci. USA *96*, 6353–6358.

Chang, M.K., Binder, C.J., Miller, Y.I., Subbanagounder, G., Silverman, G.J., Berliner, J.A., and Witztum, J.L. (2004). Apoptotic cells with oxidation-specific epitopes are immunogenic and proinflammatory. J. Exp. Med. *200*, 1359–1370.

Chen, G.Y., Tang, J., Zheng, P., and Liu, Y. (2009). CD24 and Siglec-10 selectively repress tissue damage-induced immune responses. Science *323*, 1722–1725.

Chen, G.Y., Chen, X., King, S., Cavassani, K.A., Cheng, J., Zheng, X., Cao, H., Yu, H., Qu, J., Fang, D., et al. (2011). Amelioration of sepsis by inhibiting sialidase-mediated disruption of the CD24-SiglecG interaction. Nat. Biotechnol. *29*, 428–435.

Chen, W., Han, C., Xie, B., Hu, X., Yu, Q., Shi, L., Wang, Q., Li, D., Wang, J., Zheng, P., et al. (2013). Induction of Siglec-G by RNA viruses inhibits the innate immune response by promoting RIG-I degradation. Cell *152*, 467–478.

Chou, M.Y., Fogelstrand, L., Hartvigsen, K., Hansen, L.F., Woelkers, D., Shaw, P.X., Choi, J., Perkmann, T., Bäckhed, F., Miller, Y.I., et al. (2009). Oxidation-specific epitopes are dominant targets of innate natural antibodies in mice and humans. J. Clin. Invest. *119*, 1335–1349.

Ding, C., Liu, Y., Wang, Y., Park, B.K., Wang, C.Y., Zheng, P., and Liu, Y. (2007). Siglecg limits the size of B1a B cell lineage by down-regulating NFkappaB activation. PLoS ONE *2*, e997.

Fillatreau, S., Sweenie, C.H., McGeachy, M.J., Gray, D., and Anderton, S.M. (2002). B cells regulate autoimmunity by provision of IL-10. Nat. Immunol. *3*, 944–950.

Hilgendorf, I., Theurl, I., Gerhardt, L.M., Robbins, C.S., Weber, G.F., Gonen, A., Iwamoto, Y., Degousee, N., Holderried, T.A., Winter, C., et al. (2014). Innate response activator B cells aggravate atherosclerosis by stimulating T helper-1 adaptive immunity. Circulation *129*, 1677–1687.

Hoffmann, A., Kerr, S., Jellusova, J., Zhang, J., Weisel, F., Wellmann, U., Winkler, T.H., Kneitz, B., Crocker, P.R., and Nitschke, L. (2007). Siglec-G is a B1 cell-inhibitory receptor that controls expansion and calcium signaling of the B1 cell population. Nat. Immunol. *8*, 695–704.

Horie, K., Miyata, T., Maeda, K., Miyata, S., Sugiyama, S., Sakai, H., van Ypersole de Strihou, C., Monnier, V.M., Witztum, J.L., and Kurokawa, K. (1997). Immunohistochemical colocalization of glycoxidation products and lipid peroxidation products in diabetic renal glomerular lesions. Implication for glycoxidative stress in the pathogenesis of diabetic nephropathy. J. Clin. Invest. *100*, 2995–3004.

Hotamisligil, G.S. (2006). Inflammation and metabolic disorders. Nature *444*, 860–867.

Hutzler, S., Özgör, L., Naito-Matsui, Y., Kläsener, K., Winkler, T.H., Reth, M., and Nitschke, L. (2014). The ligand-binding domain of Siglec-G is crucial for its selective inhibitory function on B1 cells. J. Immunol. *192*, 5406–5414.

Imai, Y., Kuba, K., Neely, G.G., Yaghubian-Malhami, R., Perkmann, T., van Loo, G., Ermolaeva, M., Veldhuizen, R., Leung, Y.H., Wang, H., et al. (2008). Identification of oxidative stress and Toll-like receptor 4 signaling as a key pathway of acute lung injury. Cell *133*, 235–249.

Ishida, D., Su, L., Tamura, A., Katayama, Y., Kawai, Y., Wang, S.F., Taniwaki, M., Hamazaki, Y., Hattori, M., and Minato, N. (2006). Rap1 signal controls B cell receptor repertoire and generation of self-reactive B1a cells. Immunity *24*, 417–427.

Jellusova, J., Düber, S., Gückel, E., Binder, C.J., Weiss, S., Voll, R., and Nitschke, L. (2010). Siglec-G regulates B1 cell survival and selection. J. Immunol. *185*, 3277–3284.

Khoo, L.H., Thiam, C.H., Soh, S.Y., and Angeli, V. (2015). Splenic extrafollicular reactions and BM plasma cells sustain IgM response associated with hypercholesterolemia. Eur. J. Immunol. *45*, 1300–1312.

Kyaw, T., Tay, C., Khan, A., Dumouchel, V., Cao, A., To, K., Kehry, M., Dunn, R., Agrotis, A., Tipping, P., et al. (2010). Conventional B2 B cell depletion ameliorates whereas its adoptive transfer aggravates atherosclerosis. J. Immunol. *185*, 4410–4419.

Kyaw, T., Tay, C., Krishnamurthi, S., Kanellakis, P., Agrotis, A., Tipping, P., Bobik, A., and Toh, B.H. (2011). B1a B lymphocytes are atheroprotective by secreting natural IgM that increases IgM deposits and reduces necrotic cores in atherosclerotic lesions. Circ. Res. *109*, 830–840.

Lewis, M.J., Malik, T.H., Ehrenstein, M.R., Boyle, J.J., Botto, M., and Haskard, D.O. (2009). Immunoglobulin M is required for protection against atherosclerosis in low-density lipoprotein receptor-deficient mice. Circulation *120*, 417–426.

Libby, P., Ridker, P.M., and Hansson, G.K. (2011). Progress and challenges in translating the biology of atherosclerosis. Nature *473*, 317–325.

Ma, Z., Choudhury, A., Kang, S.A., Monestier, M., Cohen, P.L., and Eisenberg, R.A. (2008). Accelerated atherosclerosis in ApoE deficient lupus mouse models. Clin. Immunol. *127*, 168–175.

Miller, Y.I., Choi, S.H., Wiesner, P., Fang, L., Harkewicz, R., Hartvigsen, K., Boullier, A., Gonen, A., Diehl, C.J., Que, X., et al. (2011). Oxidation-specific epitopes are danger-associated molecular patterns recognized by pattern recognition receptors of innate immunity. Circ. Res. *108*, 235–248.

Müller, J., Lunz, B., Schwab, I., Acs, A., Nimmerjahn, F., Daniel, C., and Nitschke, L. (2015). Siglec-G deficiency leads to autoimmunity in aging C57BL/6 mice. J. Immunol. *195*, 51–60.

Notkins, A.L. (2004). Polyreactivity of antibody molecules. Trends Immunol. *25*, 174–179.

Ogden, C.A., Kowalewski, R., Peng, Y., Montenegro, V., and Elkon, K.B. (2005). IGM is required for efficient complement mediated phagocytosis of apoptotic cells in vivo. Autoimmunity *38*, 259–264.

Pao, L.I., Lam, K.P., Henderson, J.M., Kutok, J.L., Alimzhanov, M., Nitschke, L., Thomas, M.L., Neel, B.G., and Rajewsky, K. (2007). B cell-specific deletion of protein-tyrosine phosphatase Shp1 promotes B-1a cell development and causes systemic autoimmunity. Immunity *27*, 35–48.

Perry, H.M., Bender, T.P., and McNamara, C.A. (2012). B cell subsets in atherosclerosis. Front. Immunol. *3*, 373.

Pfrengle, F., Macauley, M.S., Kawasaki, N., and Paulson, J.C. (2013). Copresentation of antigen and ligands of Siglec-G induces B cell tolerance independent of CD22. J. Immunol. *191*, 1724–1731.

Roman, M.J., and Salmon, J.E. (2007). Cardiovascular manifestations of rheumatologic diseases. Circulation *116*, 2346–2355.

Rosenfeld, S.M., Perry, H.M., Gonen, A., Prohaska, T.A., Srikakulapu, P., Grewal, S., Das, D., Mcskimming, C., Taylor, A.M., Tsimikas, S., et al. (2015). B-1b cells secrete atheroprotective IgM and attenuate atherosclerosis. Circ. Res. *117*, e28–39.

Sage, A.P., Tsiantoulas, D., Baker, L., Harrison, J., Masters, L., Murphy, D., Loinard, C., Binder, C.J., and Mallat, Z. (2012). BAFF receptor deficiency reduces the development of atherosclerosis in mice–brief report. Arterioscler. Thromb. Vasc. Biol. *32*, 1573–1576.

Stewart, C.R., Stuart, L.M., Wilkinson, K., van Gils, J.M., Deng, J., Halle, A., Rayner, K.J., Boyer, L., Zhong, R., Frazier, W.A., et al. (2010). CD36 ligands promote sterile inflammation through assembly of a Toll-like receptor 4 and 6 heterodimer. Nat. Immunol. *11*, 155–161.

Tabas, I. (2010). Macrophage death and defective inflammation resolution in atherosclerosis. Nat. Rev. Immunol. *10*, 36–46.

Tall, A.R., and Yvan-Charvet, L. (2015). Cholesterol, inflammation and innate immunity. Nat. Rev. Immunol. *15*, 104–116.

Tsiantoulas, D., Gruber, S., and Binder, C.J. (2012). B-1 cell immunoglobulin directed against oxidation-specific epitopes. Front. Immunol. *3*, 415.

Tsiantoulas, D., Diehl, C.J., Witztum, J.L., and Binder, C.J. (2014). B cells and humoral immunity in atherosclerosis. Circ. Res. *114*, 1743–1756.

Tsiantoulas, D., Perkmann, T., Afonyushkin, T., Mangold, A., Prohaska, T.A., Papac-Milicevic, N., Millischer, V., Bartel, C., Hörkkö, S., Boulanger, C.M., et al. (2015). Circulating microparticles carry oxidation-specific epitopes and are recognized by natural IgM antibodies. J. Lipid Res. *56*, 440–448.

Uhlar, C.M., and Whitehead, A.S. (1999). Serum amyloid A, the major vertebrate acute-phase reactant. Eur. J. Biochem. *265*, 501–523.

Walenbergh, S.M., Koek, G.H., Bieghs, V., and Shiri-Sverdlov, R. (2013). Non-alcoholic steatohepatitis: the role of oxidized low-density lipoproteins. J. Hepatol. *58*, 801–810.

Weber, C., and Noels, H. (2011). Atherosclerosis: current pathogenesis and therapeutic options. Nat. Med. *17*, 1410–1422.

Winer, D.A., Winer, S., Chng, M.H., Shen, L., and Engleman, E.G. (2014). B Lymphocytes in obesity-related adipose tissue inflammation and insulin resistance. Cell. Mol. Life Sci. *71*, 1033–1043.

Zouggari, Y., Ait-Oufella, H., Bonnin, P., Simon, T., Sage, A.P., Guérin, C., Vilar, J., Caligiuri, G., Tsiantoulas, D., Laurans, L., et al. (2013). B lymphocytes trigger monocyte mobilization and impair heart function after acute myocardial infarction. Nat. Med. *19*, 1273–1280.

Cell Reports, Volume *14*

Supplemental Information

Sialic Acid-Binding Immunoglobulin-like Lectin G Promotes Atherosclerosis and Liver Inflammation by Suppressing the Protective Functions of B-1 Cells

Sabrina Gruber, Tim Hendrikx, Dimitrios Tsiantoulas, Maria Ozsvar-Kozma, Laura Göderle, Ziad Mallat, Joseph L. Witztum, Ronit Shiri-Sverdlov, Lars Nitschke, and Christoph J. Binder

SUPPLEMENTAL INFORMATION

Figure S1.

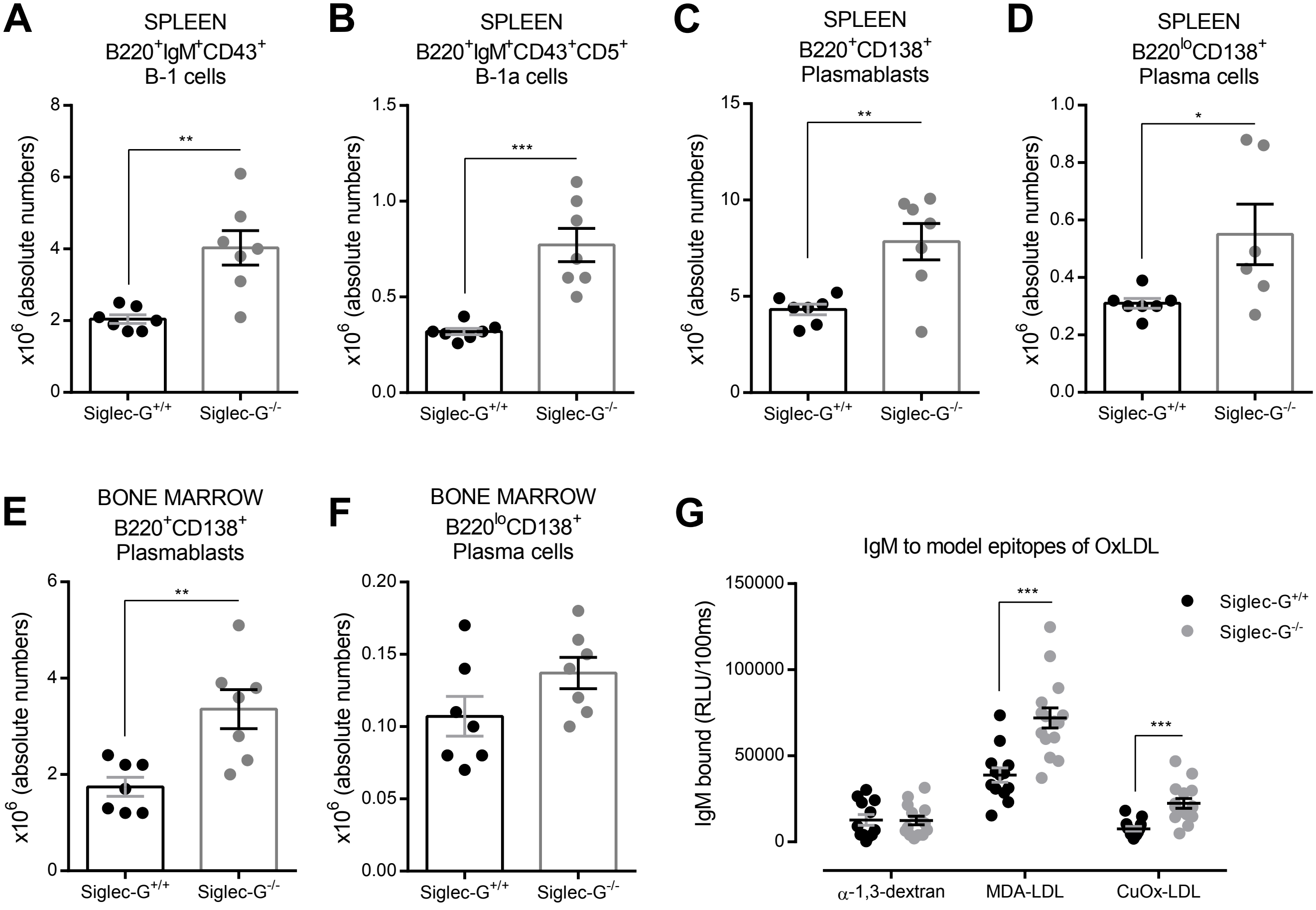

Figure S2.

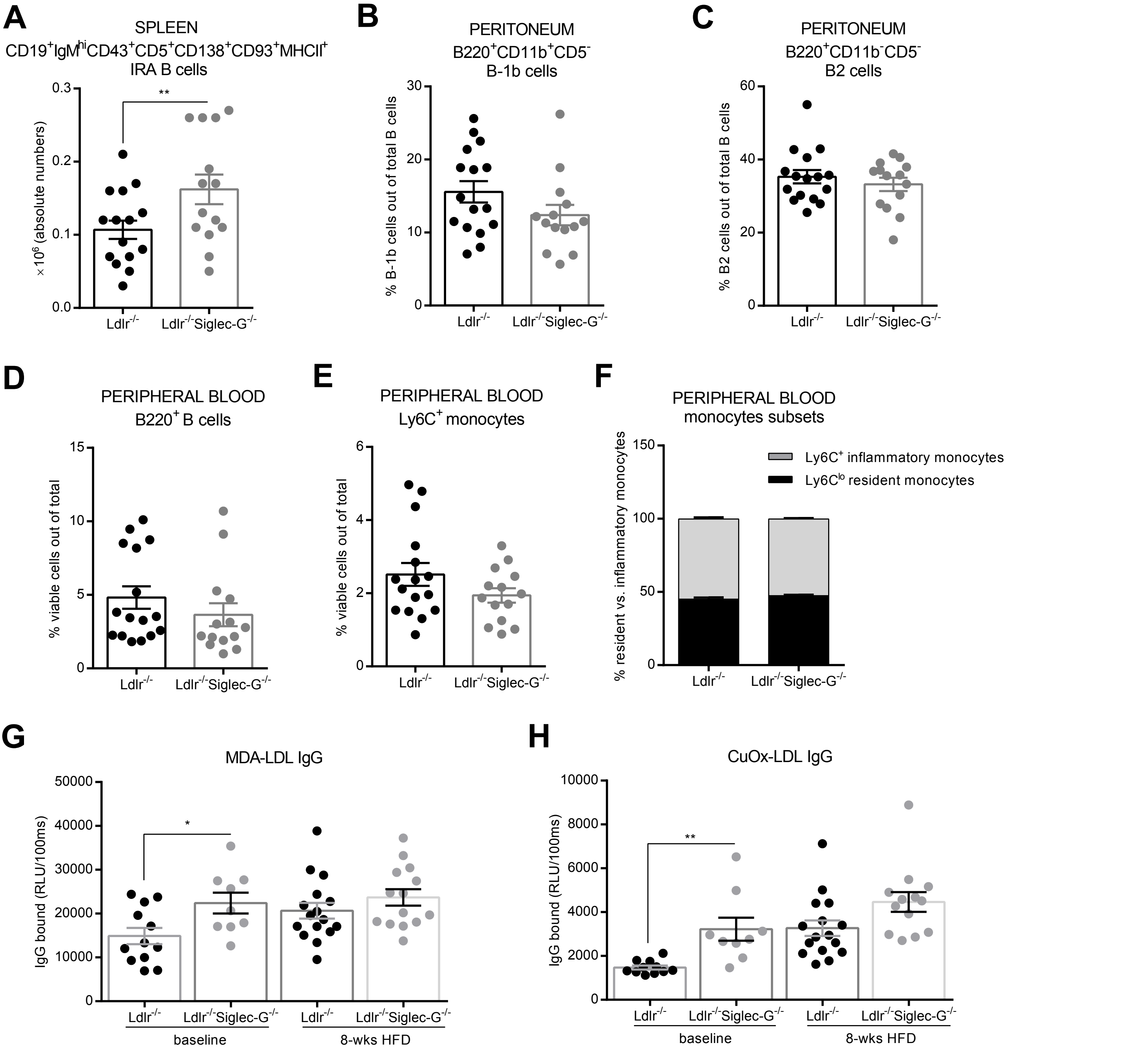
A
SPLEEN
CD19+IgMhiCD43+CD5+CD138+CD93+MHCII+
IRA B cells
×10^6 (absolute numbers)
**
Ldlr-/-
Ldlr-/-Siglec-G-/-
B
PERITONEUM
B220+CD11b+CD5-
B-1b cells
% B-1b cells out of total B cells
C
PERITONEUM
B220+CD11b-CD5-
B2 cells
% B2 cells out of total B cells
D
PERIPHERAL BLOOD
B220+ B cells
% viable cells out of total
E
PERIPHERAL BLOOD
Ly6C+ monocytes
% viable cells out of total
F
PERIPHERAL BLOOD
monocytes subsets
% resident vs. inflammatory monocytes
Ly6C+ inflammatory monocytes
Ly6Clo resident monocytes
G
MDA-LDL IgG
IgG bound (RLU/100ms)
*
baseline
8-wks HFD
H
CuOx-LDL IgG
IgG bound (RLU/100ms)
**

Figure S3.

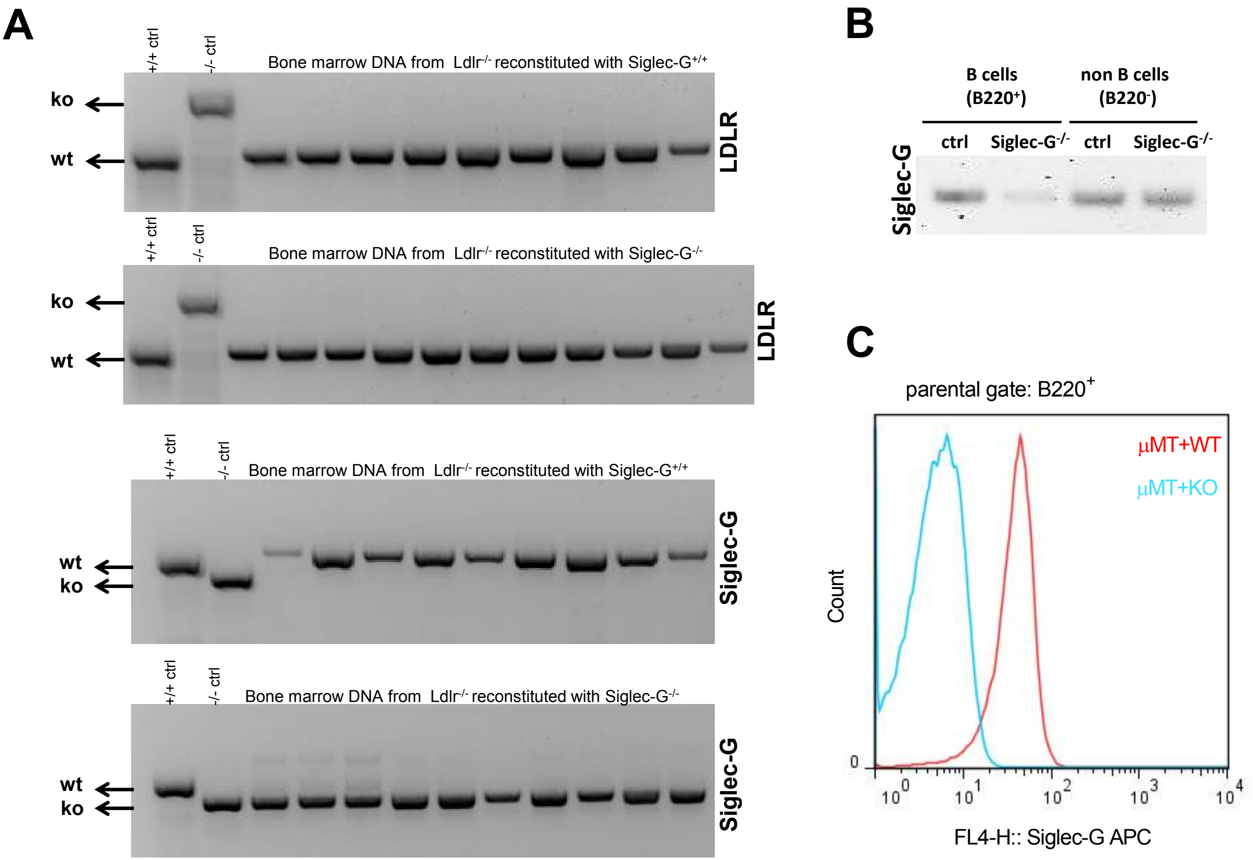
A
+/+ ctrl
-/- ctrl
Bone marrow DNA from Ldlr-/- reconstituted with Siglec-G+/+
ko
wt
LDLR
+/+ ctrl
-/- ctrl
Bone marrow DNA from Ldlr-/- reconstituted with Siglec-G-/-
ko
wt
LDLR
+/+ ctrl
-/- ctrl
Bone marrow DNA from Ldlr-/- reconstituted with Siglec-G+/+
wt
ko
Siglec-G
+/+ ctrl
-/- ctrl
Bone marrow DNA from Ldlr-/- reconstituted with Siglec-G-/-
wt
ko
Siglec-G
B
B cells (B220+)
non B cells (B220-)
ctrl
Siglec-G-/-
ctrl
Siglec-G-/-
Siglec-G
C
parental gate: B220+
μMT+WT
μMT+KO
Count
0
10^0
10^1
10^2
10^3
10^4
FL4-H:: Siglec-G APC

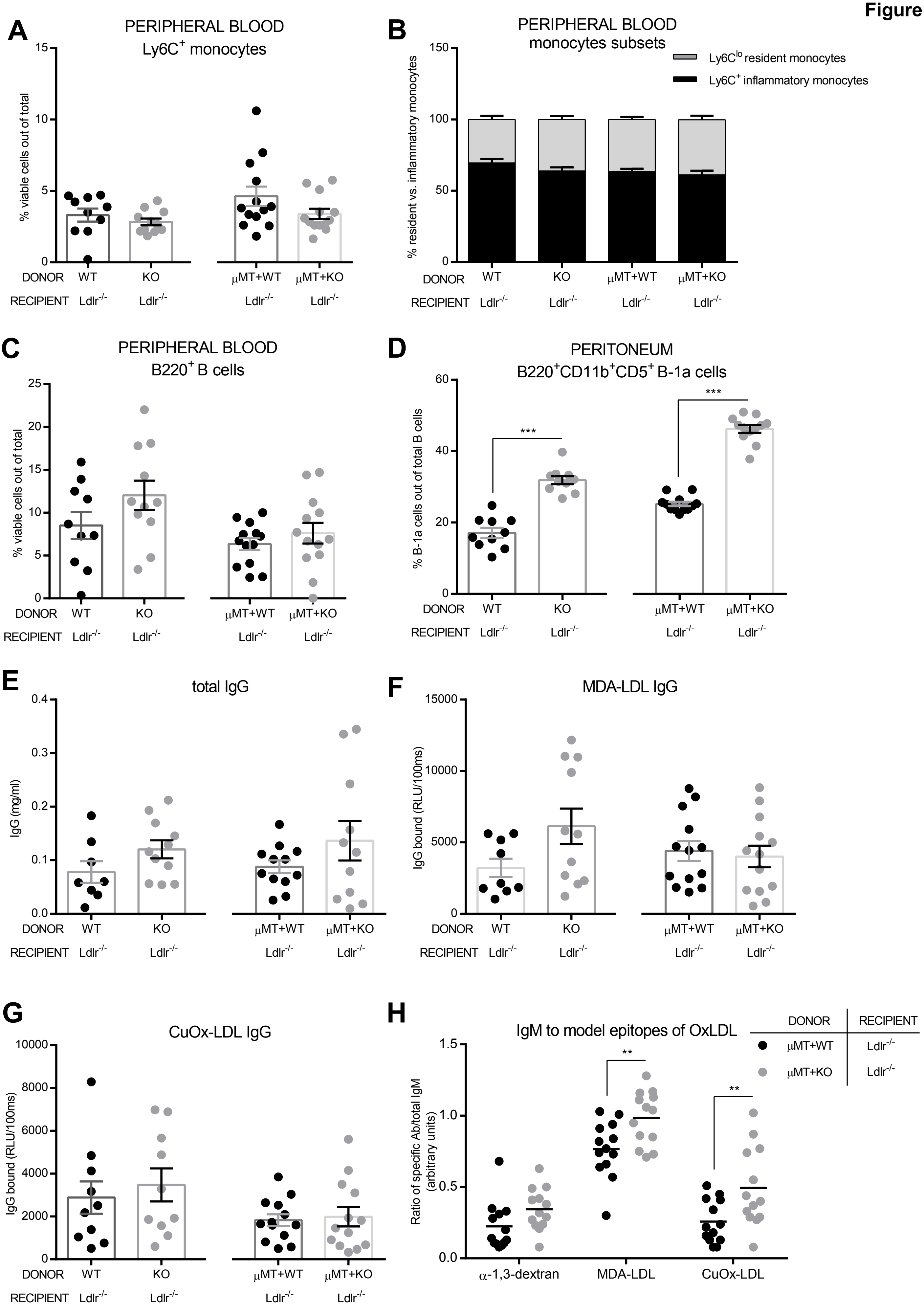
A
PERIPHERAL BLOOD
Ly6C+ monocytes
% viable cells out of total
B
PERIPHERAL BLOOD
monocytes subsets
Ly6Clo resident monocytes
Ly6C+ inflammatory monocytes
% resident vs. inflammatory monocytes
C
PERIPHERAL BLOOD
B220+ B cells
% viable cells out of total
D
PERITONEUM
B220+CD11b+CD5+ B-1a cells
% B-1a cells out of total B cells
E
total IgG
IgG (mg/ml)
F
MDA-LDL IgG
IgG bound (RLU/100ms)
G
CuOx-LDL IgG
IgG bound (RLU/100ms)
H
IgM to model epitopes of OxLDL
Ratio of specific Ab/total IgM (arbitrary units)
α-1,3-dextran
MDA-LDL
CuOx-LDL
DONOR
RECIPIENT
WT
KO
μMT+WT
μMT+KO
Ldlr-/-

Figure S5.

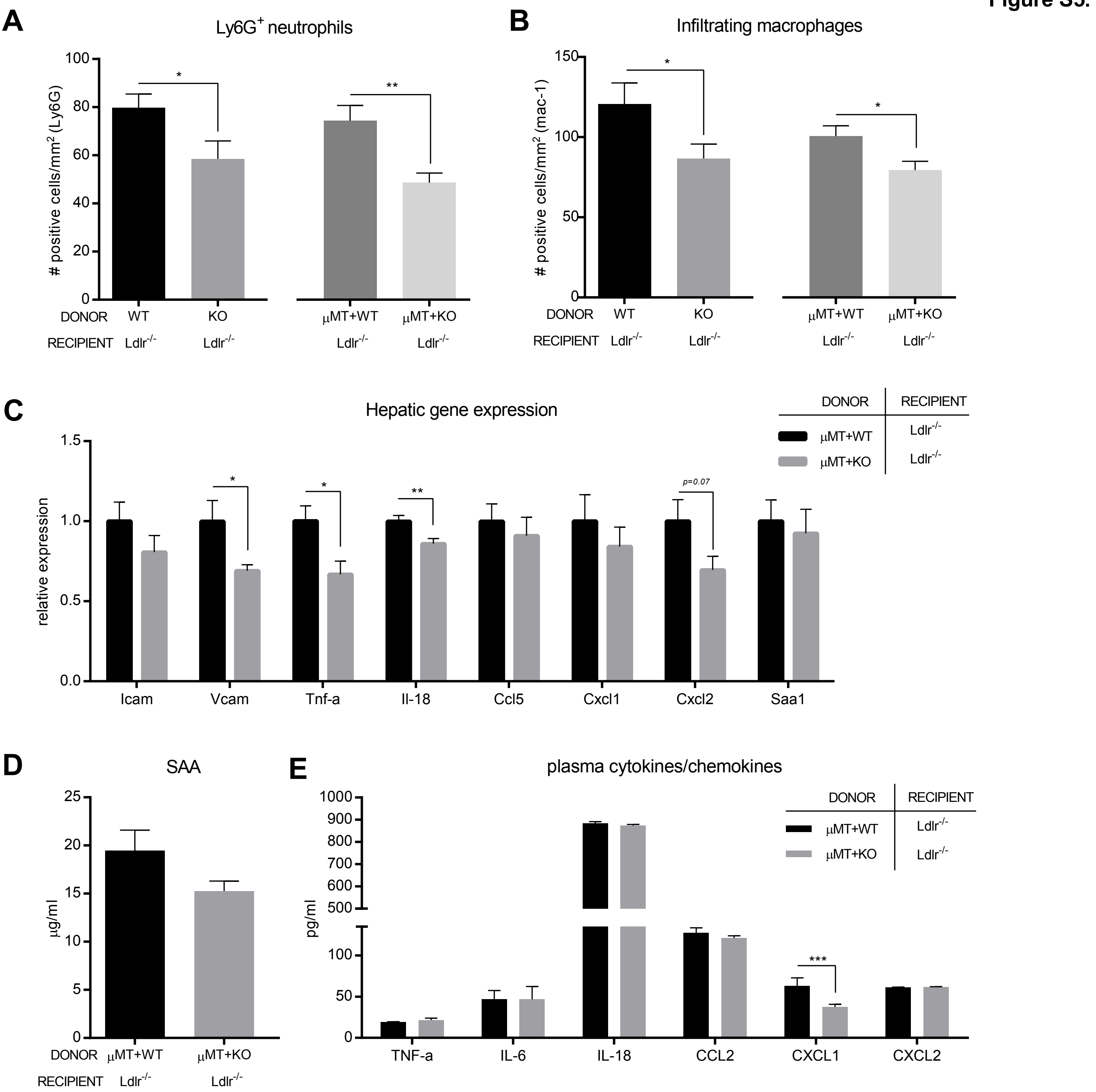
A
Ly6G+ neutrophils
positive cells/mm2 (Ly6G)
100
80
60
40
20
0
*
**
DONOR
WT
KO
μMT+WT
μMT+KO
RECIPIENT
Ldlr-/-
B
Infiltrating macrophages
positive cells/mm2 (mac-1)
150
100
50
0
*
*
DONOR
WT
KO
μMT+WT
μMT+KO
RECIPIENT
Ldlr-/-
C
Hepatic gene expression
relative expression
1.5
1.0
0.5
0.0
*
*
**
p=0.07
Icam
Vcam
Tnf-a
Il-18
Ccl5
Cxcl1
Cxcl2
Saa1
DONOR
RECIPIENT
μMT+WT
μMT+KO
Ldlr-/-
D
SAA
μg/ml
25
20
15
10
5
0
DONOR
μMT+WT
μMT+KO
RECIPIENT
Ldlr-/-
E
plasma cytokines/chemokines
pg/ml
1000
900
800
700
600
500
100
50
0
***
TNF-a
IL-6
IL-18
CCL2
CXCL1
CXCL2
DONOR
RECIPIENT
μMT+WT
μMT+KO
Ldlr-/-

Figure S6.

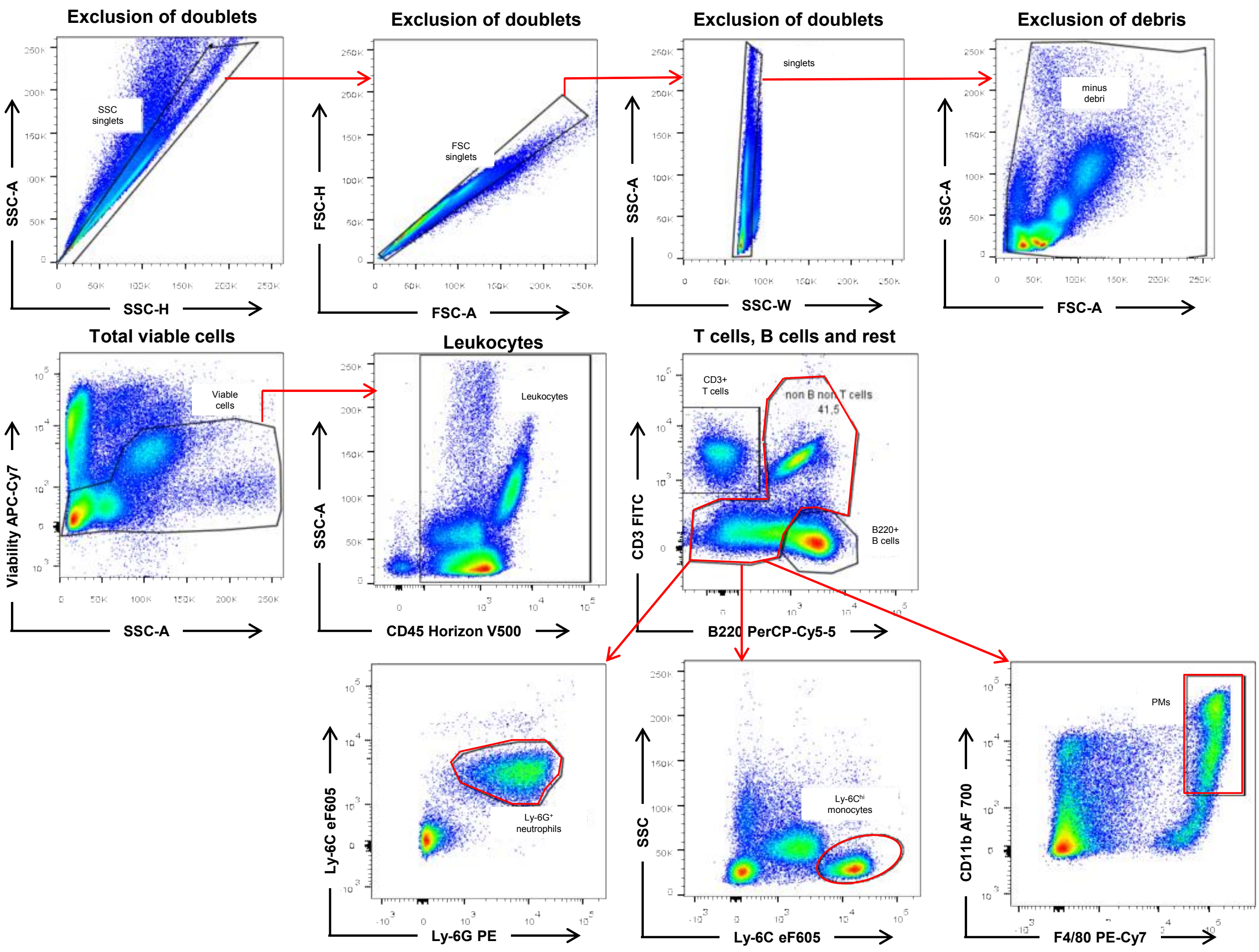

Exclusion of doublets
SSC singlets
SSC-A
SSC-H
Exclusion of doublets
FSC singlets
FSC-H
FSC-A
Exclusion of doublets
singlets
SSC-A
SSC-W
Exclusion of debris
minus debri
SSC-A
FSC-A
Total viable cells
Viable cells
Viability APC-Cy7
SSC-A
Leukocytes
Leukocytes
SSC-A
CD45 Horizon V500
T cells, B cells and rest
CD3+ T cells
non B non T cells
41,5
B220+ B cells
CD3 FITC
B220 PerCP-Cy5-5
Ly-6G+ neutrophils
Ly-6C eF605
Ly-6G PE
Ly-6Chi monocytes
SSC
Ly-6C eF605
PMs
CD11b AF 700
F4/80 PE-Cy7

Figure S7.

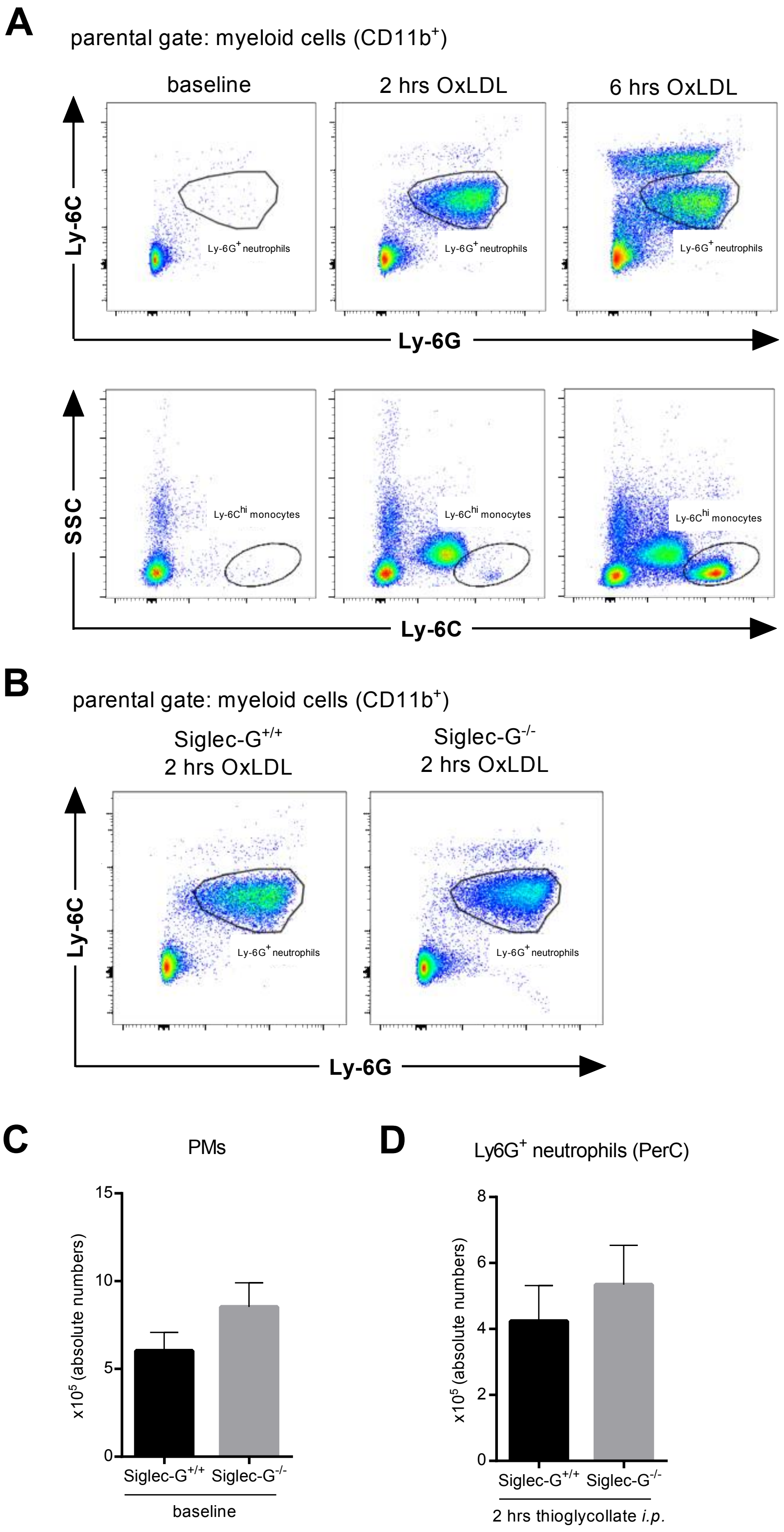
A
parental gate: myeloid cells (CD11b+)
baseline
2 hrs OxLDL
6 hrs OxLDL
Ly-6C
Ly-6G+ neutrophils
Ly-6G
SSC
Ly-6Chi monocytes
Ly-6C
B
parental gate: myeloid cells (CD11b+)
Siglec-G+/+
2 hrs OxLDL
Siglec-G-/-
2 hrs OxLDL
Ly-6C
Ly-6G+ neutrophils
Ly-6G
C
PMs
x10^5 (absolute numbers)
0
5
10
15
Siglec-G+/+
Siglec-G-/-
baseline
D
Ly6G+ neutrophils (PerC)
x10^5 (absolute numbers)
0
2
4
6
8
Siglec-G+/+
Siglec-G-/-
2 hrs thioglycollate i.p.

SUPPLEMENTAL FIGURE LEGENDS

Figure S1. Characterization of natural IgM responses in C57BL/6 Siglec-G-deficient mice, Related to Figure 1. (A) Absolute numbers of $B220^{+}IgM^{+}CD43^{+}$ B-1 cells from the spleen. (B) Absolute numbers of $B220^{+}IgM^{+}CD43^{+}CD5^{+}$ B-1a cells from the spleen. (C) Absolute numbers of $B220^{+}CD138^{+}$ plasmablasts from the spleen. (D) Absolute numbers of $B220^{lo}CD138^{+}$ plasma cells from the spleen. (E) Absolute numbers of $B220^{+}CD138^{+}$ plasmablasts from the bone marrow. (F) Absolute numbers of $B220^{lo}CD138^{+}$ plasma cells from the bone marrow. (G) IgM antibody binding to α-1,3-dextran, MDA-LDL, and CuOx-LDL in plasma was determined by ELISA. Plasma was diluted 1:100 and 1:500 and antibody binding was measured in triplicates. Shown are data of *Siglec-G*$^{+/+}$ *and Siglec-G*$^{-/-}$ mice at 8-12 weeks of age. Symbols represent individual mice. Horizontal bars represent the mean and error bars represent SEM of each group. * $P \leq 0.05$, ** $P \leq 0.01$, *** $P \leq 0.001$.

Figure S2. Immunological characterization of *Ldlr*$^{-/-}$ and *Ldlr*$^{-/-}$*Siglec-G*$^{-/-}$ after 8 weeks of atherogenic diet feeding, Related to Figure 1. (A) Absolute numbers of splenic $CD19^{+}IgM^{hi}CD43^{+}CD5^{+}CD138^{+}$ $CD93^{+}MHCII^{+}$ innate response activator (IRA) B cells. (B and C) Relative numbers of peritoneal cavity $B220^{+}CD5^{-}CD11b^{+}$ B-1b cells (B) and $B220^{+}CD5^{-}CD11b^{-}$ B2 cells (C) out of total B cells. (D) Relative numbers of peripheral blood $B220^{+}$ B cells. (E) Relative numbers of peripheral blood $Ly6C^{+}$ monocytes. (F) Relative numbers of peripheral blood $Ly6C^{+}$ inflammatory and $Ly6C^{lo}$ resident monocytes. (G-H) Titers of MDA-LDL IgG (G) and CuOx-LDL IgG (H) in plasma of *Ldlr*$^{-/-}$ and *Ldlr*$^{-/-}$*Siglec-G*$^{-/-}$ mice were determined by ELISA at baseline and after 8 weeks of atherogenic diet. Plasma samples were diluted 1:100 and 1:500 and antibody binding was measured in triplicates. Symbols represent individual mice (14-16 mice per group). Horizontal bars represent the mean and error bars represent SEM of each group. * $P \leq 0.05$, ** $P \leq 0.01$, *** $P \leq 0.001$.

Figure S3. Successful bone marrow reconstitution in *Ldlr*$^{-/-}$ recipient mice, Related to Figure 3. *Ldlr*$^{-/-}$ mice were reconstituted with bone marrow cells isolated from either *Siglec-G*$^{+/+}$ mice or *Siglec-G*$^{-/-}$ [KO] mice or a mixture of 80% bone marrow from *µMT* and 20% either *Siglec-G*$^{+/+}$ [WT] or *Siglec-G*$^{-/-}$ [KO] fed an atherogenic diet for 10 weeks. (A) At time of sacrifice bone marrow cells were collected from mice reconstituted with *Siglec-G*$^{+/+}$ [WT] and *Siglec-G*$^{-/-}$ [KO] and genomic DNA was extracted and amplified for Ldlr and Siglec-G genes. (For some mice DNA could not be recovered successfully). Tail DNA from *Siglec-G*$^{+/+}$, *Siglec-G*$^{-/-}$ and *Ldlr*$^{-/-}$ mice were used as positive and negative controls. (B) Validation of B cell restricted Siglec-G deficiency in *Ldlr*$^{-/-}$ mice after reconstitution with µMT+WT and µMT+*Siglec-G*$^{-/-}$ [KO] bone marrow after 10 weeks of atherogenic diet. DNA of sorted splenic B ($B220^{+}$) and non-B cells ($B220^{-}$) was isolated and pooled samples were analyzed by PCR for the Siglec-G gene. (C) Flow cytometry for Siglec-G expression. Representative histogram of Siglec-G expression on

$B220^+$ B cells in the spleens of mice reconstituted with µMT+WT and µMT+KO bone marrow after 10 weeks of atherogenic diet.

Figure S4. Immunological characterization of bone marrow chimeric *Ldlr*$^{-/-}$ mice after 10 weeks of atherogenic diet, Related to Figure 3. (A) Relative numbers of peripheral blood $Ly6C^+$ monocytes. (B) Relative numbers of peripheral blood $Ly6C^+$ inflammatory and $Ly6C^{lo}$ resident monocytes. (C) Relative numbers of peripheral blood $B220^+$ B cells. (D) Relative numbers of $B220^+CD5^+CD11b^+$ B-1a cells out of total B cells from the peritoneal cavities. (E) Quantification of total IgG antibodies in plasma. Plasma samples were diluted 1:70,000 and measured in triplicates. (F-G) Binding of IgG antibodies to (F) MDA-LDL and (G) CuOx-LDL was determined by ELISA. Plasma samples were diluted between 1:100 and 1:500 and antibody binding was measured in triplicates. Data are expressed as RLU per 100 ms. Symbols represent individual mice. (H) IgM antibody repertoire to model epitopes of OxLDL. Titers of α-1,3-dextran IgM, MDA-LDL IgM and CuOx-LDL IgM in plasma were determined by ELISA. Plasma was diluted 1:100 and 1:500 and antibody binding was measured in triplicates. The data is plotted as the ratio of antigen-specific IgM to total IgM. Shown are data of *Ldlr*$^{-/-}$ mice reconstituted with *Siglec-G*$^{+/+}$ (n = 10) vs. *Siglec-G*$^{-/-}$ [KO] (n = 11) and µMT+*Siglec-G*$^{+/+}$ (n = 13) vs. µMT+*Siglec-G*$^{-/-}$ [KO] (n = 13) bone marrow after 10 weeks of atherogenic diet. Horizontal bars represent the mean and error bars represent SEM of each group. * $P \leq 0.05$, ** $P \leq 0.01$, *** $P \leq 0.001$.

Figure S5. Selective Siglec-G deficiency in B cells protects from atherogenic diet-induced hepatic and systemic inflammation, Related to Figure 4. (A, B) Quantification of infiltrating neutrophils (A) and macrophages (B) in liver sections of *Ldlr*$^{-/-}$ mice reconstituted with *Siglec-G*$^{+/+}$ [WT] vs. *Siglec-G*$^{-/-}$ [KO] and µMT+*Siglec-G*$^{+/+}$ [WT] vs. µMT+*Siglec-G*$^{-/-}$ [KO] and fed with an atherogenic diet for 10 weeks. Sections were stained with anti-Ly6G and anti-mac-1 antibody, and positively stained cells were counted and expressed as number of positive cells per mm^2. (C) Relative gene expression of *Icam, Vcam, Tnf-α, Il-18, Ccl5, Cxcl1, Cxcl2* and *Saa1* mRNA in livers of *Ldlr*$^{-/-}$ mice reconstituted with µMT+*Siglec-G*$^{+/+}$ [WT] vs. µMT+*Siglec-G*$^{-/-}$ [KO] bone marrow and fed an atherogenic diet for 10 weeks. The expression of individual genes was normalized to the house-keeping gene S12 and expressed relatively to the expression in µMT+*Siglec-G*$^{+/+}$ [WT] control mice. (D) Quantification of SAA levels in plasma of *Ldlr*$^{-/-}$ mice reconstituted with µMT+*Siglec-G*$^{+/+}$ [WT] vs. µMT+*Siglec-G*$^{-/-}$ [KO] bone marrow and fed an atherogenic diet for 10 weeks. Plasma samples were diluted 1:300 and measured in triplicates. (E) Quantification of a panel of chemokines and cytokines in plasma of *Ldlr*$^{-/-}$ mice reconstituted with µMT+*Siglec-G*$^{+/+}$ [WT] vs. µMT+*Siglec-G*$^{-/-}$ [KO] bone marrow and fed an atherogenic diet for 10 weeks by multiplex assay. Plasma samples were diluted 1:2. Data represent mean ± SEM of 9-13 mice per group. * $P \leq 0.05$, ** $P \leq 0.01$, *** $P \leq 0.001$.

Figure S6. Representative flow cytometry plots and gating strategy for analysis of peritoneal cell populations upon sterile peritonitis induction, Related to Figure 5. *Siglec-G*$^{+/+}$ and *Siglec-G*$^{-/-}$ mice were injected *i.p.* with OxLDL (25 µg/g body weight) and peritoneal exudate cells were harvested to monitor infiltration of immune cells. Representative flow cytometry plots and gating strategy is shown: After exclusion of doublets and cell debris by FSC and SSC, viable cells were identified by staining with a viability dye. CD45 was used to separate individual cell populations. T cells and B cells were identified by CD3 and B220 markers, respectively. The "non T and non B cells" gate (CD3^{-}B220^{-} cells) was further used to define Ly-6G^{+} neutrophils, Ly-6C^{hi} monocytes and CD11b^{+}F4/80^{+} macrophages.

Figure S7. Characterization of sterile peritonitis in Siglec-G-deficient mice, Related to Figure 5. (A) Representative flow cytometry plots for Ly6G^{+} neutrophils and Ly6G^{hi} inflammatory monocytes of C57BL/6 mice are shown at baseline, as well as 2, 6, and 12 hours after OxLDL injection. (B) Representative flow cytometry plots for Ly6G^{+} neutrophils in the PLF of *Siglec-G*$^{+/+}$ vs. *Siglec-G*$^{-/-}$ mice 2 hours after *i.p.* OxLDL injection are shown. (C) Absolute numbers of CD11b^{+}F4/80^{+} peritoneal macrophages (PMs) in the peritoneal lavage fluid at baseline in *Siglec-G*$^{+/+}$ and *Siglec-G*$^{-/-}$ mice. (D) Absolute numbers of Ly6G^{+} neutrophils in the peritoneal lavage fluid after 2 hours of thioglycollate injection (25 µl/g body weight, *i.p.*) in *Siglec-G*$^{+/+}$ and *Siglec-G*$^{-/-}$ mice. Data represent mean and error bars represent SEM of 3-7 mice per group. * $P \leq 0.05$, ** $P \leq 0.01$, *** $P \leq 0.001$.

SUPPLEMENTAL EXPERIMENTAL PROCEDURES

Bone marrow transplantation

Bone marrow transplantation studies were performed as previously described (Binder et al., 2003, Fillatreau et al., 2002, Sage et al., 2012). Ten weeks-old male *Ldlr*$^{-/-}$ mice received a single dose of 9.5 Gy lethal irradiation and were subsequently injected intravenously via the retro-orbital plexus with 1×10^7 bone marrow cells from either *Siglec-G*$^{-/-}$ ($n = 12$) or *Siglec-G*$^{+/+}$ ($n = 12$) mice. In a separate experiment in order to assess the effects of selective deficiency of Siglec-G on B cells, irradiated *Ldlr*$^{-/-}$ mice were reconstituted with a mixture of 80% bone marrow from *µMT* mice (no B cells due to disruption of the membrane exon of the mu heavy chain gene) and 20% bone marrow from either *Siglec-G*$^{+/+}$ (n = 13) or *Siglec-G*$^{-/-}$ (n = 13) mice. After 4 weeks of recovery, mice were fed an atherogenic diet containing 21% milk fat and 0.2% cholesterol (Ssniff Spezialdiäten GmbH, Soest, Germany) for 10 weeks to induce lesion formation. Three mice (two in the *Siglec-G*$^{+/+}$ and one in *Siglec-G*$^{-/-}$) died during the study.

Evaluation of atherosclerosis

The extent of atherosclerosis was assessed in the entire aorta and in cross-sections of the aortic origin, as previously described (Cardilo-Reis et al., 2012). Histological sections of the paraffin-embedded aortic origin were stained with

a modified elastic-trichrome stain for quantification of lesion size and size of necrotic areas. Macrophage areas were assessed by immunohistochemistry using anti-mouse-mac-3 antibodies (rat anti-mouse, clone M3/84, BD-Biosciences Pharmingen, San Diego, California, USA). For the quantification of lesional collagen content, sections were stained with Sirius Red (Direct Red 80, Sigma Aldrich). For the presence of IgM, immunohistochemistry was performed using an antibody against mouse IgM, µ-chain specific (Sigma-Aldrich). For indicated experimental studies, innominate arteries were isolated and embedded in paraffin as described (Reardon et al., 2003). All photographed images were quantified using Adobe Photoshop CS5 and analyzed using ImageJ 1.47 software.

Flow cytometry

At time of sacrifice, peritoneal exudates cells (PECs) were collected by lavaging the peritoneum with 10 ml of sterile HBSS + 2% FBS. PECs were pelleted; cells were counted and subsequently processed for flow cytometry. Spleens and bone marrow were harvested and passed through a cell strainer to obtain a single cell suspension and red blood cells were lysed using a commercial lysis buffer (Morphisto). Total white blood cells were isolated from blood collected in EDTA-tubes via the vena cava. Blood was diluted 1:1 in a solution of PBS containing 2% Dextran (Sigma-Aldrich) and incubated for 40 min at 37ºC to separate the red blood cells. The supernatant was harvested and used for flow cytometry analysis. 1×10^6 cells were used for flow cytometry staining, and 5×10^6 cells were lysed in 350 µl RLT RNA lysis buffer (Qiagen) and stored at -20°C.

For flow cytometric analyses, cells were blocked with anti-mouse CD16/32 blocking Ab (anti-mouse CD16/CD32, clone 93, 0.5 µg/0.5×10^6 cells, eBioscience) for 20 min at 4°C and stained for 30 min with following antibodies: PerCP-Cy5.5-labeled anti-CD45R (B220) (clone RA3-6B2, 1:800, eBioscience), FITC-labeled anti-CD23 (clone B3B4, 1:600, BD Biosciences – Pharmingen), APC-labeled anti-IgM (clone II/41, 1:600, eBioscience), phycoerythrin (PE)-labeled anti-CD43 (clone S7, 1:600, BD Biosciences – Pharmingen), biotin-labeled anti-CD21/CD35 (CR2/CR1) (clone 7E9, 1:200, BioLegend), PE-labeled anti-CD3e (clone 145-2C11, 1:400, eBioscience), FITC-labeled anti-CD4 (clone GK1.5, 1:400, eBioscience), APC-labeled anti-CD8a (clone 53-6.7, 1:400, eBioscience), FITC-labeled anti-kappa (clone 197.1, 1:400, BD Biosciences – Pharmingen), Biotin-labeled anti-lambda (clone RML-42, 1:400, BioLegend), PE-labeled anti-IgD (clone 11-26c (11-26), 1:200, eBioscience), PE-labeled anti-CD93 (clone AA4.1, 1:400, eBioscience), APC-labeled anti-Siglec-G (clone SH2.1, 1:400, eBioscience), APC-labeled anti-CD11b/Mac-1 (clone M1/70, 1:600, eBioscience), FITC-labeled anti CD11b (clone M1/70, 1:600, eBioscience), PE-labeled anti-CD5 (clone 53-7.3, 1:100, eBioscience), FITC-labeled anti-Ly6C (clone HK14, 1:200, BioLegend), PE-labeled anti-Ly6G (clone 1A8, 1:2000, BioLegend), V500-labeled anti-CD45 (clone 30-F11, 1:200, BD Biosciences – Pharmingen), BV570-labeled anti-Ly-6C (clone HK1.4, 1:100, BioLegend), eFluor 780-labeled fixable viability dye (1:1000, eBioscience), AF700-labeled anti-CD11b (clone M1/70, 1:700, eBioscience), Brilliant Violet 570-labelled anti-CD19 (1:100, clone 6D5, BioLegend), PE-labelled anti-GM-CSF (1:100, clone MP1-22E9, BioLegend), PECy7-labelled anti CD93 (1:100, clone AA4.1, eBioscience), biotin-labeled anti-CD5 (Ly-1) (1:200, clone 53-7.3, BD Pharmingen), AF700-labelled anti-MHCII (1:100, clone M5/114.15.2, eBioscience), FITC-labeled anti-IgD (1:800, clone 11-26c, eBioscience), biotin-labeled anti-CD138 (Syndecan 1) (1:200, clone 281-2, BioLegend). All stains were performed in 100 µl of FACS buffer (PBS + 10% FCS) for 30 min

at 4°C in darkness, followed by two washing steps. Stained cell populations were analyzed by multiparameter flow cytometry using a BD FACSCalibur (BD Bioscience, Franklin Lakes, New Jersey, USA) or BD FACS Fortessa, respectively. Either 1×10^5 or 1×10^6 cells per sample were stained and acquired. Dead cells and doublets were excluded by forward- and side-scatter and data were analyzed using the FlowJo version 10 data analysis software (Tree Star Inc., Oregon Corporation, Ashland, USA).

Clinical chemistry and lipid analyses

At time of sacrifice, blood was collected via the vena cava into EDTA tubes (MiniCollect® 1 ml K_3EDTA Blood Collection Tube, Greiner Bio-One), centrifuged for 30 min at 1000 rpm, and aliquots of plasma were stored at -80°C for further analyses. Total plasma cholesterol and triglycerides were measured by enzymatic methods using an automated analyzer AU5400 – Chemistry System (Beckman Coulter, Brea, California, USA). Liver lipid levels were measured by enzymatic methods using the CHOD-PAP assay (Roche Diagnostics, Basel, Switzerland) for cholesterol and a triglyceride determination kit (Sigma-Aldrich). Non-esterified fatty acids (NEFA) in the liver were determined using the NEFA-C kit (ACS-ACOD method; Wako Chemicals, Neuss, Germany). All measurements were done according to the manufacturer's protocols using a Benchmark 550 Micro-plate Reader (Bio-Rad, Hercules, CA). Protein content was measured with the bicinchoninic acid (BCA) method (Pierce, Rockford, IL) according to manufacturer’s protocol. Data are expressed as µg lipid per µg protein.

ALT levels were measured in plasma (30 µl, 1:3 diluted in physiological NaCl) of each individual mouse by use of the Reflotron system in combination with the strips for alanine aminotransferase (Roche Diagnostics, Almere, The Netherlands) according to the manufacturer's instructions.

Gene expression analysis

Liver. Total RNA was isolated from mouse liver tissue and was reversely transcribed using the iScript cDNA synthesis kit (Bio-Rad, Hercules, USA). Quantitative real-time PCR was performed using SensiMix™ SYBR green with ROX dye (Bioline**,** Randolph, USA) on an ABI7900HT instrument (Applied Biosystems). The relative expression level of target genes was determined with the LinRegPCR software (http://www.hfrc.nl, (Ruijter et al., 2009), and obtained values were normalized to the housekeeping gene ribosomal protein S12 (Rps12). Values are expressed as the relative expression compared to the control group.

Primer sequences:

Cxcl2-forward: 5’-AGTGAACTGCGCTGTCAATGC-3’;

Cxcl2-reverse: 5’-AGGCAAACTTTTTGACCGCC-3’;

TNF-α-forward: 5’-CATCTTCTCAAAATTCGAGTGACAA-3’;

TNF-α-reverse: 5’-TGGGAGTAGACAAGGTACAACCC-3’;

Cxcl1-forward: 5’-TGCACCCAAACCGAAGTCAT-3’;

Cxcl1-reverse: 5’-TTGTCAGAAGCCAGCGTTCAC-3’;

SAA1-forward: 5’-GGCTGCTGAGAAAATCAGTGATG-3’;

SAA1-reverse: 5’-TCAGCAATGGTGTCCTCATGTC-3’;

VCAM-forward: 5’-GTGTTGAGCTCTGTGGGTTTTG-3’;

VCAM-reverse: 5’-TTAATTACTGGATCTTCAGGGAATGAG-3’;

ICAM-forward: 5’-CTACCATCACCGTGTATTCGTTTC-3’;

ICAM-reverse: 5’-CGGTGCTCCACCATCCA-3’;

IL-18-forward: 5’-ACAACTTTGGCCGACTTCAC-3’;

IL-18-reverse: 5’-GGGTTCACTGGCACTTTGAT-3’;

CCL5-forward: 5’-GGAGTATTTCTACACCAGCAGCAA-3’;

CCL5-reverse: 5’-GCGGTTCCTTCGAGTGACA-3’;

S12-forward: 5’-GGAAGGCATAGCTGCTGGAGGTGT-3’;

S12-reverse: 5’-CCTTCGATGACATCCTTGGCCTGAG-3’.

Validation of successful BMT

At time of sacrifice, bone marrow cells from all mice were collected and genomic DNA was extracted by incubating cells in 40 µl of lysis buffer (1 M Tris/HCl, pH 8.0, 500 mM EDTA, 10% SDS, 2 M NaCl, 0.4 mg/ml Proteinase K) at 55°C overnight with gentle mixing, followed by 10 min incubation at 99°C to inactivate Proteinase K. The purified genomic DNA was diluted 1:10 in H_2O and amplified by PCR for the Ldlr and Siglec-G gene to identify successful bone marrow reconstitution with donor bone marrow. In order to assess selective Siglec-G deficiency on B cells, B cells and non B cells of splenocytes from recipient mice were separated using anti-CD45R (B220) MicroBeads (Milteny Biotec) according to the manufacturer’s protocol. Collected cell fractions were lysed in RLT Plus buffer (Qiagen) and stored at -20°C. Genomic DNA was purified with the AllPrep DNA/RNA Micro Kit (Qiagen) according to the manufacturer’s protocol and equal amounts (50 ng) were used for PCR to amplify the genes of interest.

Primer sequences:

Siglec-G-forward: 5'-CCGCTCGAGATGTTGTCCCGCGGGTGGTTTCA-3';

Siglec-G-reverse: 5'-CCGGAATTCCTTCCTCTTGAGAGAACCTTTGTTC-3';

Ldlr-forward: 5'-ACCCCAAGACGTGCTCCCAGGATG-3';

Ldlr-reverse: 5'-CGCAGTGCTCCTCATCTGACTTGT-3';

neo cassette primer: 5'-AGGTGAGATGACAGGAGA-3'.

SUPPLEMENTAL REFERENCES

BINDER, C. J., HORKKO, S., DEWAN, A., CHANG, M. K., KIEU, E. P., GOODYEAR, C. S., SHAW, P. X., PALINSKI, W., WITZTUM, J. L. & SILVERMAN, G. J. 2003. Pneumococcal vaccination decreases atherosclerotic lesion formation: molecular mimicry between Streptococcus pneumoniae and oxidized LDL. *Nature medicine,* 9**,** 736-43.

CARDILO-REIS, L., GRUBER, S., SCHREIER, S. M., DRECHSLER, M., PAPAC-MILICEVIC, N., WEBER, C., WAGNER, O., STANGL, H., SOEHNLEIN, O. & BINDER, C. J. 2012. Interleukin-13 protects from atherosclerosis and modulates plaque composition by skewing the macrophage phenotype. *EMBO molecular medicine,* 4**,** 1072-86.

FILLATREAU, S., SWEENIE, C. H., MCGEACHY, M. J., GRAY, D. & ANDERTON, S. M. 2002. B cells regulate autoimmunity by provision of IL-10. *Nature immunology,* 3**,** 944-50.

REARDON, C. A., BLACHOWICZ, L., LUKENS, J., NISSENBAUM, M. & GETZ, G. S. 2003. Genetic background selectively influences innominate artery atherosclerosis: immune system deficiency as a probe. *Arteriosclerosis, thrombosis, and vascular biology,* 23**,** 1449-54.

RUIJTER, J. M., RAMAKERS, C., HOOGAARS, W. M., KARLEN, Y., BAKKER, O., VAN DEN HOFF, M. J. & MOORMAN, A. F. 2009. Amplification efficiency: linking baseline and bias in the analysis of quantitative PCR data. *Nucleic acids research,* 37**,** e45.

SAGE, A. P., TSIANTOULAS, D., BAKER, L., HARRISON, J., MASTERS, L., MURPHY, D., LOINARD, C., BINDER, C. J. & MALLAT, Z. 2012. BAFF receptor deficiency reduces the development of atherosclerosis in mice--brief report. *Arteriosclerosis, thrombosis, and vascular biology,* 32**,** 1573-6.
